# Post-transcriptional Regulation of Keratinocyte Progenitor Cell Expansion, Differentiation and Hair Follicle Regression by *miR-22*

**DOI:** 10.1371/journal.pgen.1005253

**Published:** 2015-05-28

**Authors:** Shukai Yuan, Feifei Li, Qingyong Meng, Yiqiang Zhao, Lei Chen, Hongquan Zhang, Lixiang Xue, Xiuqing Zhang, Christopher Lengner, Zhengquan Yu

**Affiliations:** 1 State Key Laboratories for Agrobiotechnology, College of Biological Sciences, China Agricultural University, Beijing, China; 2 Department of Biochemistry and Molecular Biology, Basic Medical College, Tianjin Medical University, Tianjin, China; 3 Department of Animal Science, Southwest University, Rongchang, Chongqing, China; 4 Chongqing Academy of Animal Science, Rongchang, Chongqing, China; 5 Laboratory of Molecular Cell Biology and Tumor Biology, Department of Anatomy, Histology and Embryology, Beijing, China; 6 College of Food Science and Nutritional Engineering, China Agricultural University, Beijing, China; 7 Department of Animal Biology, School of Veterinary Medicine, University of Pennsylvania, Philadelphia, Pennsylvania, United States of America; 8 Institute for Regenerative Medicine, University of Pennsylvania, Philadelphia, Pennsylvania, United States of America; Stanford University School of Medicine, United States of America

## Abstract

Hair follicles (HF) undergo precisely regulated recurrent cycles of growth, cessation, and rest. The transitions from anagen (growth), to catagen (regression), to telogen (rest) involve a physiological involution of the HF. This process is likely coordinated by a variety of mechanisms including apoptosis and loss of growth factor signaling. However, the precise molecular mechanisms underlying follicle involution after hair keratinocyte differentiation and hair shaft assembly remain poorly understood. Here we demonstrate that a highly conserved microRNA, *miR-22* is markedly upregulated during catagen and peaks in telogen. Using gain- and loss-of-function approaches *in vivo*, we find that *miR-22* overexpression leads to hair loss by promoting anagen-to-catagen transition of the HF, and that deletion of *miR-22* delays entry to catagen and accelerates the transition from telogen to anagen. Ectopic activation of *miR-22* results in hair loss due to the repression a hair keratinocyte differentiation program and keratinocyte progenitor expansion, as well as promotion of apoptosis. At the molecular level, we demonstrate that *miR-22* directly represses numerous transcription factors upstream of phenotypic keratin genes, including *Dlx3*, *Foxn1*, and *Hoxc13*. We conclude that *miR-22* is a critical post-transcriptional regulator of the hair cycle and may represent a novel target for therapeutic modulation of hair growth.

## Author Summary

Up to 60% people suffer from hair loss throughout their lifetime. Hair growth undergoes recurrent cycling of growth, regression, and resting phases with a defined periodicity. The main cause of human hair loss is due to the premature transition from growth to regression. Understanding of the molecular basis underlying hair regression is important to elucidate the mechanisms of hair loss. Here, we demonstrated that *miR-22*, a highly conserved microRNA, is critical for the transition from growth to regression of the hair follicle. Importantly, *miR-22* could be a novel target for therapeutic therapy of hair loss disorders.

## Introduction

Hair follicles undergo recurrent cycles of growth (anagen), regression (catagen), and resting (telogen) phases with a defined periodicity [1,2]. Abnormal regulation of genes associated with hair cycling may cause several types of human hair growth disorders [3]. For example, male pattern baldness is due to the premature transition from anagen-to-catagen induced by androgens [4]. The hair follicle contains epithelial cells of the outer root sheath (ORS), matrix, the inner root sheath and hair shaft, and mesenchymal cells of dermal papilla [1,5]. The interaction between epithelial and mesenchymal cells is required for proper hair development and follicle cycling [2]. At the onset of growth (anagen), the dermal papilla is at the proximal end of the follicle, in close proximity to the hair stem cell niche known as the follicle bulge [6]. Signals from the condensed dermal papilla induce proliferation of hair bulge stem cells, resulting in the downward extension of the hair germ where it ultimately envelops the dermal papilla and triggers hair matrix formation. Transit-amplifying matrix cells proliferate rapidly in response to signals from the dermal papilla after which they terminally differentiate to form the inner root sheath and hair shaft. Several important signaling pathways including Wnt and BMP play an important role in hair differentiation [7]. Further, a host of transcription factors promote hair differentiation during anagen including *Gata3* [8], *Cutl1* [9], *Lef1* [10], *Dlx3* [11], *Msx2* [12], *Foxn1* [13], *TCF3* [14] and *Hoxc13* [15]. In contrast to anagen, catagen is the physiological involution of the hair follicle. The hair follicle rapidly degenerates and shortens until it is again adjacent to the hair follicle stem cell reservoir in the bulge region. This process may be triggered by a variety of stimuli, including apoptosis and loss of supportive growth factor signaling needed to maintain cell proliferation and differentiation during anagen [16]. While some apoptotic triggers promoting catagen have been defined, including Bcl2/Bax [17], p53 [18], p57 [19], and the transforming growth factors, TGF-β1 and TGF-β2 [20,21], little is known about how the stimulatory signals that drive anagen are terminated upon entry to catagen and maintained during telogen.

MicroRNAs play important roles in many biological processes including hair follicle development [22], and hundreds of microRNAs are expressed in the skin [23,24]. Global ablation of microRNA activity through genetic deletion of microRNA processing enzymes, *DGCR8*, *Drosha*, and *Dicer* demonstrated that microRNAs are critical for both embryonic and adult hair follicle development [23,25,26]. Conditional deletion of *Drosha* and *Dicer* during anagen causes failure of catagen and follicular degradation [25], indicating a critical role of microRNAs in the transition of anagen to catagen. However, the specific microRNAs involved in this process are unknown. Recently, numerous reports have documented the functional contribution of several specific microRNAs to hair development, including *miR-24* [27], *miR-125b* [28], *miR-31* [24], and *miR-205* [29], and thus a microRNA network governing hair follicle development is beginning to emerge.

The function of *miR-22* in hair follicles is of particular interest, as microRNA profiling revealed that *miR-22* expression markedly increases during the catagen stage, reaching peak expression at the transition to telogen [24], suggesting a potential role for *miR-22* in hair follicle involution. Here we utilize *miR-22* gain- and loss-of-function mouse models to investigate the physiological role of *miR-22* in hair cycling. We demonstrate that *miR-22* is an important post-transcriptional regulator that governs exit from anagen and maintenance of the hair follicle in telogen through inhibition of a transcriptional program driving keratinocyte proliferation, differentiation, and hair shaft assembly.

## Results

### *miR-22* expression pattern during hair cycling

**Fig 1:**
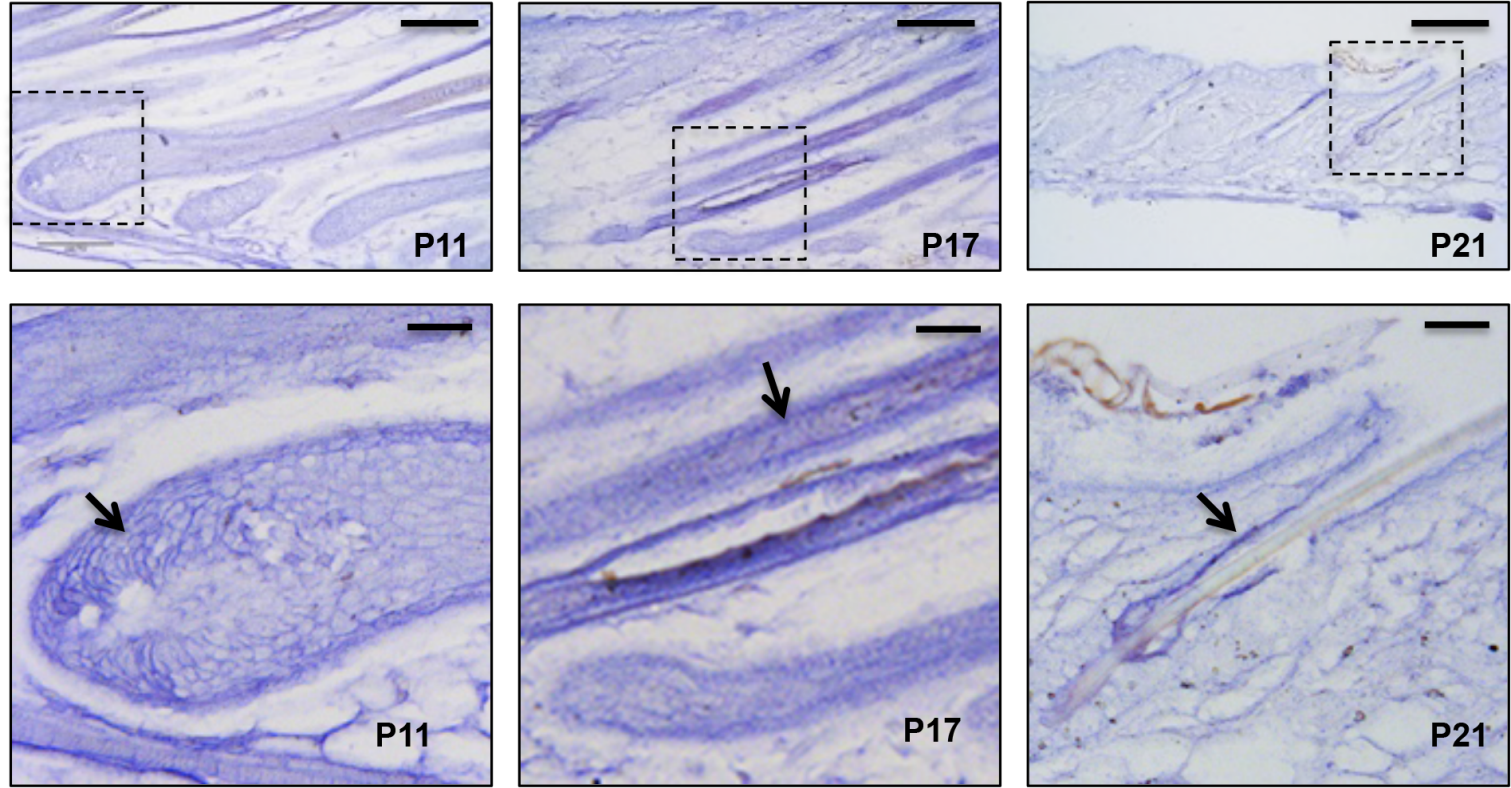
*miR-22* expression during hair cycling. *In situ* hybridization for *miR-22* in mouse backskin at P11, P17 and P21. The areas outlined by dashed boxes are shown at higher magnification in the second row. Arrows indicated *in situ* signal. Scale bars: the first rows, 100 m; the second row, 25 m.

To elucidate the role of *miR-22* in hair follicle development, we initially confirmed published findings of *miR-22* upregulation during catagen [24]. *miR-22* is markedly upregulated during catagen and reaches maximal expression in telogen (S1A Fig), suggesting a potential role of *miR-22* in hair involution. Further, *in-situ* hybridization at successive time points during anagen, catagen and telogen phases revealed that *miR-22* expressing cells are located in the ORS and hair matrix but not in the IRS at postnatal day 11 (P11, mid-anagen) (Fig 1). At P17 (catagen), *miR-22* is strongly expressed throughout the hair follicle including both ORS and IRS, with the *in-situ* signal becoming weaker in the hair matrix (Fig 1). At P21 (telogen), *miR-22* is strongly expressed in the ORS (Fig 1). At P30, the mid-anagen phase of the second hair cycle, *miR-22* is again mainly expressed in the upper ORS (S1B and S1C Fig). We observed *miR-22* signal in a few cells of hair matrix in close proximity of the dermal papilla (S1C Fig). In older animals, hair follicles reside much longer in telogen [30]. Thus, we examined the *miR-22* expression level in the skin of 18 month-old mice. As expected, *miR-22* expression increases in the aged skin (S1D Fig), consistent with a potential function for *miR-22* as a regulator of follicle regression.

### *miR-22* induction causes hair loss *in vivo*

**Fig 2:**
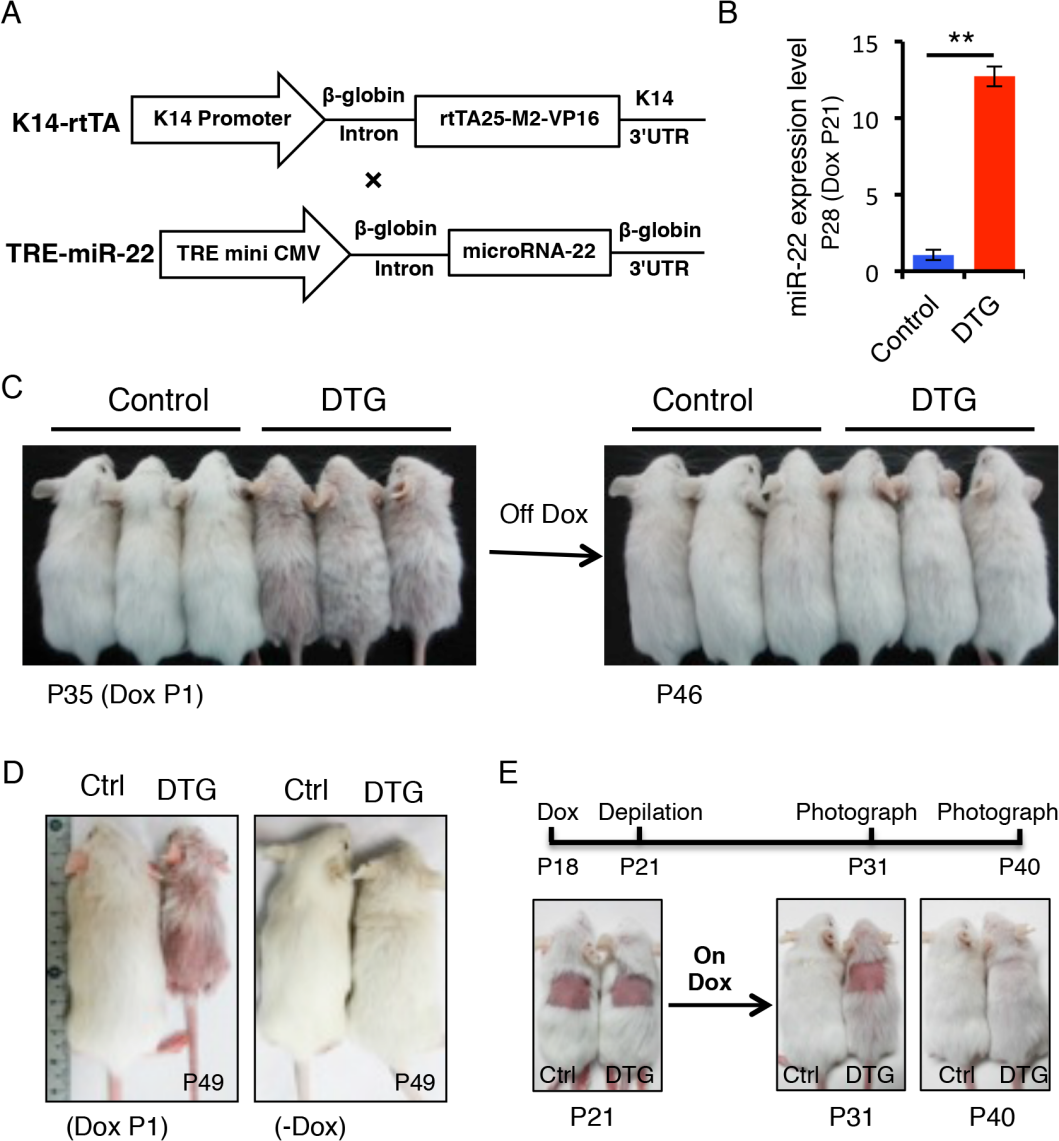
*miR-22* overexpression results in hair loss. (**A**) Schematic maps of constructs used to generate *K14-rtTA*/*TRE-miR-22* double transgenic mice (DTG). (**B**) qPCR analysis for *miR-22* showing that *miR-22* is strongly induced in the Dox-treated DTG mice. The DTG mice were treated with Dox at P21. Samples were collected at P28. ** p < 0.01. (**C**) The Dox-treated DTG mice exhibit hair loss at 35 days. Both control and DTG mice were treated by oral administration of 2 mg/mL Dox in the drinking water at P1. After removing Dox at P35, the hair loss phenotype recovers by P46. (DTG n = 3; control n = 3). (**D**) Hair loss in DTG mice at P49 when treated with Dox at P1. (**E**) External hair regrowth is delayed in the DTG mice after depilation. Both control and DTG mice were treated with Dox at P18. Hairs were plucked at P21. Photographed at P31 and P40. (DTG n = 21; control n = 16).

To determine the functional consequences of *miR-22* activity in hair follicles, we generated mice in which *miR-22* overexpression could be induced in the ORS of hair follicles with temporal specificity. *MiR-22* was placed under the control of a tetracycline regulatory element (*TRE*) (Fig 2A) and *TRE-miR-22* transgenic mice were generated. *TRE-miR-22* mice were crossed with *K14-rtTA* transgenic mice, in which the Keratin 14 (K14) promoter that is active in the basal layer of the epidermis and the ORS of the hair follicle controls expression of the reverse tetracycline transactivator (*rtTA*). After induction with Doxycycline (Dox), *K14-rtTA/TRE-miR-22* double transgenic (DTG) mice exhibited robust *miR-22* expression in the basal layer of the epidermis and ORS of the hair follicle (S1B Fig). Expression analysis demonstrated strong *miR-22* induction in DTG mice, compared to *TRE-miR22* and *K14-rtTA* controls (Fig 2B).

We initially induced *miR-22* expression at postnatal day 1 during embryonic anagen stage. Strikingly, Dox-treated DTG mice began to exhibit premature hair loss 35 days after induction (Fig 2C). Within 11 days after Dox withdrawal at P35, there was a full recovery from the hair loss phenotype (Fig 2C), demonstrating that the effect of *miR-22* induction on hair growth is reversible. When DTG mice were maintained on Dox, hair loss became more pronounced at P49 during the telogen phase, while hair growth of the control mice was normal and morphologically indistinguishable from wildtype littermates (Fig 2D). The hair loss persisted as long as the mice were maintained on Dox. In addition to the hair loss phenotype, we also found that the DTG mice were smaller in size compared to the control littermates (Fig 2C and 2D). Because the *K14* promoter is also active in the esophagus and stomach, the reduction in body size is most likely due to digestion defects caused by *miR-22* induction.

To further explore the effect of *miR-22* on hair morphorgenesis, we induced *miR-22* expression at embryonic day 15 when the hair placode begins to form. *miR-22* induction resulted in obvious thinning of the hair coat in DTG pups at P12 (S2A Fig), indicating that *miR-22* induction represses hair morphorgenesis. Surprisingly, the DTG pups died at around P17. We next initiated a synchronous hair growth cycle at P21 by depilating the dorsal hair concomitant to *miR-22* induction. External hair regrowth was observed in littermate controls 10 days post-depilation, but was absent in the depilated DTG skin (Fig 2E). Similar findings were observed at P65 when both control and DTG mice were treated with Dox at P53 and depilated at P56 (S2B Fig). Taken together, our data reveal that *miR-22* overexpression is sufficient to repress hair morphorgenesis and development *in vivo*.

### *miR-22* induction is sufficient to promote anagen-to-catagen transition of hair follicles

**Fig 3:**
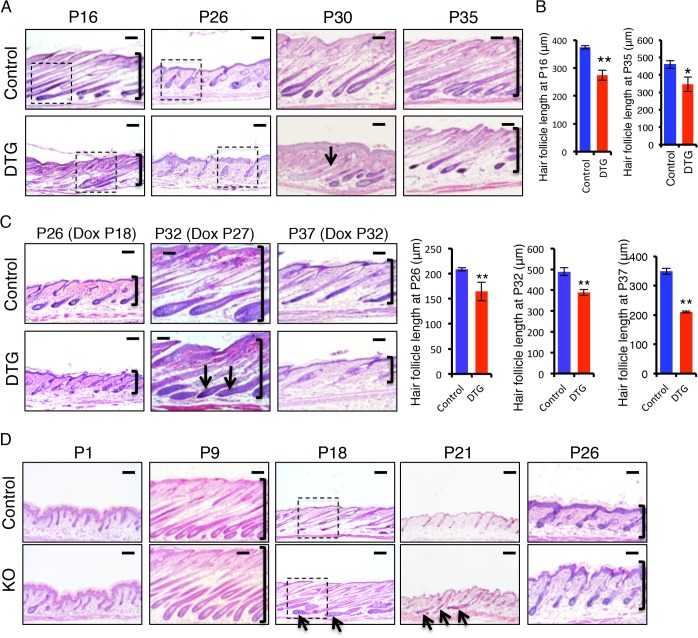
*miR-22* promotes the transition from anagen to catagen and retards the transition from telogen to anagen. (**A**) Representative histological images of dorsal skin from Dox-treated DTG mice and their Dox-treated WT or *K14-rtTA* (control) littermates at the indicated postnatal ages (P). Both control and DTG mice were treated with Dox at P1. Arrows point to hair follicles without a keratinized medulla. Brackets indicate hair follicle length. Higher magnification images indicated by dashed box shown in S2D Fig. Each time point, DTG n = 3; Control, n = 3. Scale bar, 100 m. (**B**) Quantification of hair follicle length in 3 control and 3 DTG mice at P16 and P35. ** p < 0.01; * p < 0.05. (**C**) Representative histological images of dorsal skin from control and DTG mice under conditions of short-term Dox treatment. Quantification of hair follicle length in 3 control and 3 DTG mice at P26, P32 and P37. ** p < 0.01. Brackets indicate the hair follicle length. Both control and DTG mice were treated with Dox at P18, P27 and P32 and analyzed at P26, P32 and P37, respectively. Arrows point to the regressing hair bulb. Each time point, DTG, n = 3; Control, n = 3. Scale bar, 100 m. (**D**) Representative histological images of dorsal skin from *miR-22* KO and their control (WT or heterozygous) littermates at the indicated postnatal ages. Arrows point to hair follicles exhibiting delayed regression. Brackets indicate hair follicle length. Higher magnification images indicated by dashed box are shown in S2F Fig. Each time point, KO n = 3, control n = 3. Scale bar, 100 m.

To investigate how *miR-22* overexpression impairs hair development, we studied the progression of hair cycling by analyzing histology of DTG hair follicles at successive time points after Dox induction. Both DTG and control mice were treated with Dox at P1. At P9, the DTG follicles were morphologically normal, suggesting a completion of hair follicle morphogenesis (S2C Fig). Abnormal hair follicles were clearly observed in the DTG backskin by P16. While the hair follicles of control littermates were in early catagen at P16, the DTG follicles had already entered late catagen, characterized by the narrow hair bulb and shortened hair length (Fig 3A and 3B and S2D Fig). This indicates that *miR-22* induction promotes the anagen-to-catagen transition. Both control and DTG follicles can normally enter telogen at P21, although the DTG follicles appeared slightly abnormal to be considered a typical telogen follicle (S2C Fig). By P26, control follicles entered early anagen, while the DTG follicles remained in telogen (Fig 3A and S2D Fig), suggesting that miR-22 induction retards the telogen-to-anagen transition. By P30 and 35, control follicles entered anagen and formed enlarged hair bulbs with differentiated hair shafts. While the DTG follicles also formed hair bulbs, these hair bulbs were smaller in size. The extent of downward growth in DTG follicles was reduced (Fig 3A and 3B), and the DTG follicles had no keratinized medulla (Fig 3A). By P43 and P46, the control follicles shortened and entered late catagen. While DTG hair follicles also entered catagen, characterized by narrow hair bulbs, the DTG follicles failed to further regress and shorten (S2C Fig). Most likely, the hair cycle is arrested in a mid-catagen phase, which may be the major cause of hair loss at this stage. As this phenomenon is contrary to the promotion of the anagen-to-catagen transition in the first hair cycle, we asked whether short-term *miR-22* induction would also result in an arrest of the hair cycle at this stage. We induced *miR-22* expression in mid-anagen at P27, and collected samples at P32 in late anagen. Control hair follicles were morphologically in late anagen (Fig 3C), while the DTG follicles had prematurely entered mid-catagen, characterized by narrow hair bulbs and short follicles (Fig 3C).

We next induced *miR-22* expression at P32 in late anagen, and collected samples at P37 of early catagen. We found that *miR-22* induction markedly promoted progression to catagen (Fig 3C). These findings demonstrate that *miR-22* induction promotes the transition from anagen to catagen, as well as progression through catagen. To further confirm this, we examined expression of a number of genes normally expressed in catagen at P16 (early catagen), in response to 16 days of Dox induction (S2E Fig). Expression of *MMP11*, *K16*, *TGFβ2*, *SPINK12* are markedly upregulated in DTG backskin (S2E Fig), supporting a function for *miR-22* in promoting the anagen-to-catagen transition. To test this idea that *miR-22* represses the telogen-to-anagen transition, we induced miR-22 expression at P18 and collected samples at P26. The control hair follicles normally entered anagen, while DTG follicles were much delayed (Fig 3C). The data further supported that *miR-22* retards the telogen-to-anagen transition.

In addition to defective follicular development, the DTG epidermis became markedly thickened and was hyperproliferative by P30, with increased numbers of Ki67 positive cells and P63 positive cells (S3 Fig), however differentiation of DTG epidermis appeared unaffected (S3 Fig).

### *miR-22* is required for proper anagen-to-catagen transition

The *TRE-miR-22* mouse model demonstrates the sufficiency of miR-22 in promoting the anagen-to-catagen transition, but does not address a physiological requirement in this process. We thus examined histology of *miR-22* knockout (KO) hair follicles at successive time points. No significant difference was found in the KO follicles at P1 (Fig 3D), however by P9 hair follicle length in the KO was significantly longer than in the controls, although KO follicles appeared morphologically normal (Fig 3D and S2G Fig). By P18, the defects in KO follicles became more pronounced. Control follicles had entered late catagen at this time point, however KO follicles were delayed in catagen entry, evidenced by a large hair bulb (Fig 3D and S2F Fig). By P21, control follicles uniformly had entered telogen, while up to 15% late catagen-stage follicles remained in the KO backskin, indicating a delay in hair development (Fig 3D and S2G Fig). By P26 both control and KO hair follicles entered a new anagen, however the penetrance of downward growth was increased in the KO follicles (Fig 3D and S2G Fig). Overall, the *miR-22* KO phenotype is in direct opposition to that of *miR-22* gain of function. Taken together, these data point to an important physiological role for *miR-22* in promoting the anagen-to-catagen transition and in proper telogen maintenance in hair follicles.

### *miR-22* induction inhibits keratinocyte expansion and differentiation, and promotes apoptosis

**Fig 4:**
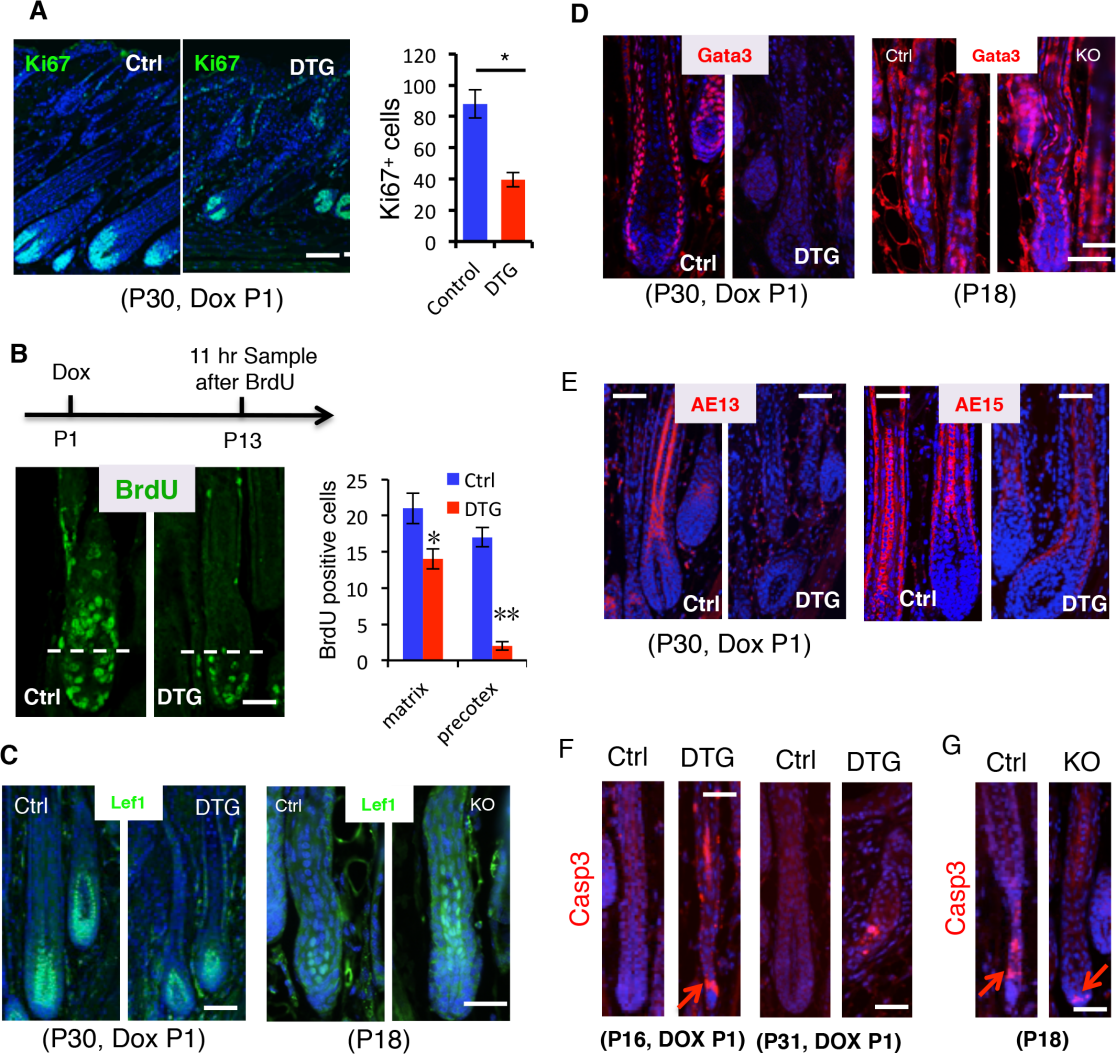
*miR-22* induction represses cell differentiation and promotes apoptosis. (**A**) Immunofluorescence for Ki67 in control and DTG hair follicles at P30. Quantification of Ki67 positive cells per follicle in control and DTG hair follicles. Scale bar, 50 m. * p < 0.05. The mice were treated with Dox at P1. (**B**) Schematic of BrdU experiment. Immunofluorescence for BrdU in control and DTG skin at 11 hours post BrdU pulse. Scale bar, 50 m. Quantification of BrdU^+^ cells per Matrix and per precotex in the control and DTG hair follicles at 11 hours post BrdU pulse. ** p < 0.01; * p < 0.05. (**C**) Immunofluorescence for Lef1 in control and DTG hair follicles at P30, and in control and *miR-22* KO hair follicles at P18. Scale bar, 50 m. (**D**) Immunofluorescence for Gata3 in control and DTG hair follicles at P30, and in control and *miR-22* KO hair follicles at P18. The control and DTG mice were treated with Dox at P1. Scale bar, 50 m. (**E**) Immunofluorescence for AE13 and AE15 in control and DTG skin at P30. The mice were treated with Dox at P1. Scale bar, 50 m. (**F**) Immunofluorescence for Cleaved Caspase 3 in control and DTG hair follicles at indicated time points. Arrows indicate positive signals. Scale bar, 50 m. (**G**) Immunofluorescence for Cleaved Caspase 3 (Casp3) in control and *miR-22* KO hair follicles at P18. Arrows indicate positive signals. Control, n = 3; KO, n = 3. Scale bar, 50 m.

To understand how *miR-22* gain of function induces hair loss, we asked whether sustained *miR-22* expression regulates cell proliferation and differentiation in the hair follicle. By P30, control follicles are in mid-anagen, with large hair bulbs and terminally differentiated hair shafts. Both DTG and control follicles were morphologically similar at P30, after 30 days of Dox administration (Fig 3A). Subsequently, the hair loss phenotype manifests in DTG mice after P30 (Fig 2C). Thus, we chose to examine proliferation and differentiation at P30, the onset of phenotypic manifestation. The number of proliferative cells was significantly reduced in the DTG matrix (Fig 4A), suggesting that *miR-22* represses cell proliferation. The hair loss defect could arise from an impairment of matrix cells in producing differentiated lineages. To test this possibility, we first examined the proliferative expansion of matrix cells. P13 mice were pulsed by a single injection of BrdU and analyzed 11 hours later. In control follicles, BrdU labeled cells could readily be observed in the hair precortex. In contrast, upward movement of BrdU^+^ cells into the DTG precotex was impaired (Fig 4B), suggesting defects in both proliferation and migration. To further pursue this, we treated DTG mice with Dox at P18, depilated hair at P21, and assessed new hair generation at P27 after 11-hour BrdU pulse. As expected, BrdU labeled cells were present in the precortex of control follicles (S4A Fig). In contrast, the DTG precortex lacked the BrdU labeled cells (S4A Fig). These data support a model in which *miR-22* inhibits the proliferative expansion and subsequent differentiation of keratinocyte progenitors.

To investigate the regulation of miR-22 on keratinocyte differentiation, we first examined transcription factors that are important for keratinocyte differentiation. *Lef1* is a transcription factor that promotes the differentiation of hair stem cells [31]. Examination of Lef1 expression in the DTG hair follicles revealed a decrease in Lef1 positive cells (Fig 4C and S4B Fig). In contrast, an increase in Lef1 positive cells was observed in the *miR-22* KO mice at P18 (Fig 4C and S4B Fig). The transcription factor *Gata-3* plays an essential role in the differentiation of IRS progenitor cells [8], and Gata-3 signals were negative in DTG follicles (Fig 4D). In contrast, Gata-3 positive cells increased in the *miR-22* KO follicles (Fig 4D and S4B Fig). The data further suggest that miR-22 represses keratinocyte differentiation. We next examined markers of terminal keratinocyte differentiation. AE13 is a hair cortex marker, which is used to detect cortical keratins. While the follicles of control littermates were AE13 positive, the DTG follicles completely lacked AE13 reactivity (Fig 4E), suggesting that *miR-22* induction inhibits hair cortex formation. Expression of the IRS and medulla tricohyalins marker AE15 was detected in both of these sites in control follicles. In contrast, AE15 immunoreactivity was reduced in the IRS of DTG follicles relative to control follicles, and DTG follicles lacked AE15 positive medulla tricohyalins (Fig 4E). These results demonstrate that *miR-22* overexpression inhibits differentiation of HF keratinocytes.

Involution of the hair follicle during catagen is driven by apoptosis [16], and thus we examined cleaved Caspase 3 in both DTG and *miR-22* KO hair follicles. DTG hair follicles at P16 and P31 after Dox induction at P1 showed an increase in apoptotic events, whereas control follicles have no apoptotic cells at these stages (Fig 4F). In contrast, *miR-22* KO follicles have significantly fewer apoptotic cells than the control follicles at P18 (Fig 4G and S4B Fig). Thus, these data demonstrate that *miR-22* also induces apoptosis of hair follicle keratinocytes, which may further contribute to the hair loss phenotype in DTG mice.

### *miR-22* induction represses colony forming capacity of hair follicle stem cells and hair neogenesis

**Fig 5:**
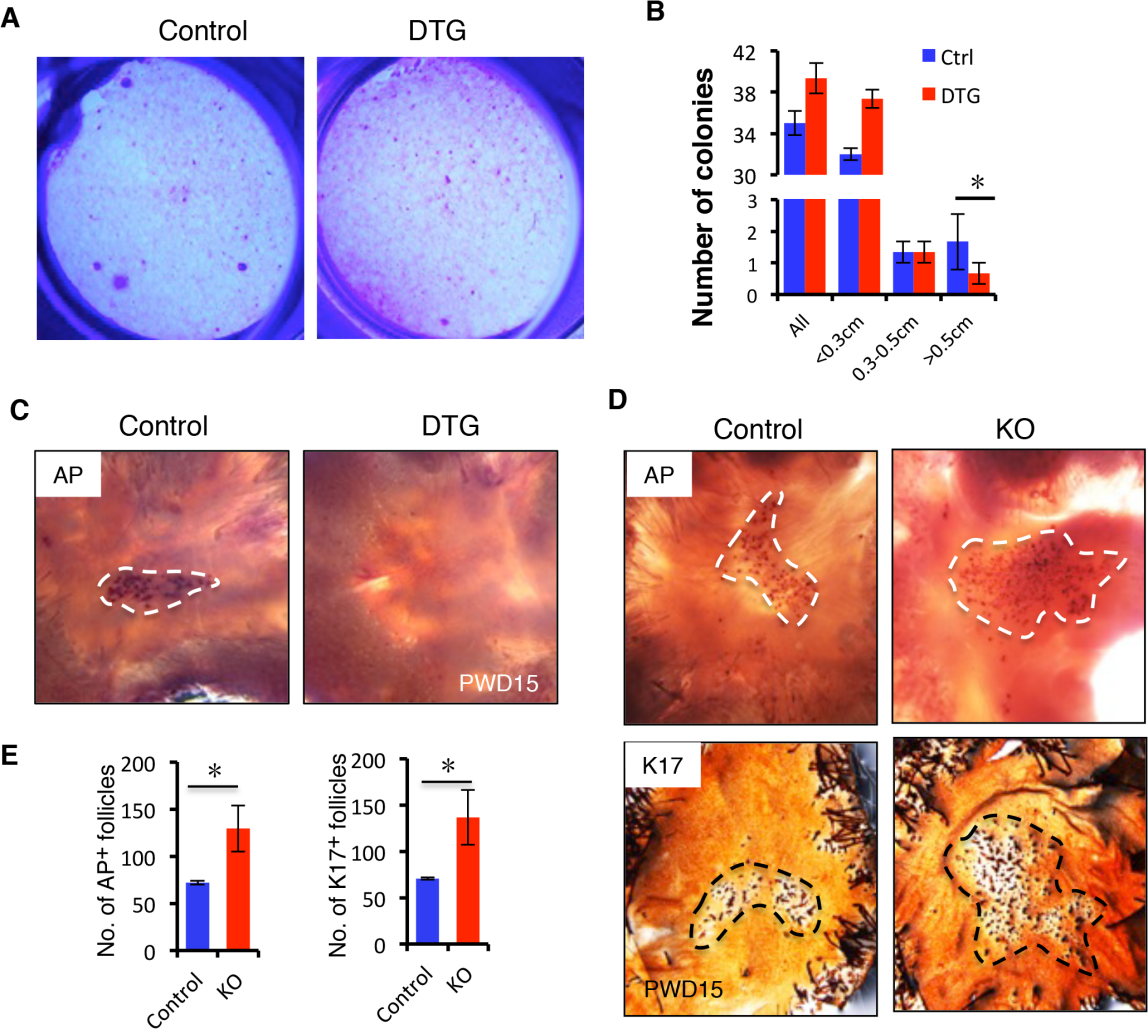
*miR-22* induction represses HFSC colony formation capacity and hair neogenesis. (**A**) The formation of large holoclones is significantly reduced in the DTG HFSCs. (**B**) Quantification of colonies of different size based on 3 independent experiments. * p < 0.05. (**C**) Newly regenerating hair stained for alkaline phosphatase activity (AP). New follicles form in the center of the wound at 15 days post wounding in the control mice, while no hair follicle are visible in the DTG mice. Dox was administered at P21. Dashed lines mark the areas of new hair follicle regeneration. Control, n = 12; DTG, n = 12. (**D**) (upper panels) Newly regenerating hairs stained for alkaline phosphatase activity (AP) in Control and miR-22 KO mice. (lower panels) Newly regenerating hairs stained for K17. Control, n = 8; KO, n = 8. (**E**) Quantification of newly regenerating hair in D based on 8 independent experiments. * p < 0.05.

The failed proliferative expansion of follicles observed upon *miR-22* overexpression indicates that *miR-22* may affect stem cell self-renewal and commitment. We examined the Sox9^+^ bulge stem cell compartment and observed no obvious difference in the frequency of hair follicle stem cells (HFSCs) in the DTG follicles (S5A Fig). Flow cytometric profiles further confirmed that the DTG and control hair follicles contained equivalent numbers of CD49f^+^, CD34^+^ HFSCs (S5B Fig). We next isolated HFSCs from control and DTG skin and compared their colony forming capacity *in vitro*. Strikingly, the number of large holoclones was significantly reduced in DTG HFSCs (Fig 5A and 5B), indicating that *miR-22* overexpression compromises colony forming capacity of hair follicle stem cells. This further explains why the DTG hair bulb is smaller in size at P30 (Fig 3A).

We next asked whether *miR-22* induction also inhibits hair follicle neogenesis following 1 cm^2^ skin wounding (S5C Fig). We induced *miR-22* expression at the time of wounding and collected samples at 15 days post-wounding (PWD). Interestingly, while a certain number of hair follicles regenerated *de novo* within the wound center of control mice, hair follicle neogenesis was completely abrogated in DTG mice (Fig 5C). In contrast, we found a significant increase number of newly formed hair follicles in *miR-22* KO mice 15 days post-wounding (Fig 5D and 5E). Taken together, these findings provide evidence that *miR-22* represses colony forming capacity of HFSCs and hair follicle neogenesis.

### *miR-22* is a critical negative regulator of the keratinocyte differentiation gene expression program during catagen and telogen

To gain mechanistic insights into the molecular events underlying transitions from anagen to catagen, to telogen, we analyzed the transcriptome profiles of catagen (mid-catagen via late anagen) and telogen (telogen via mid-catagen) [32]. The gene ontology (GO) categories of genes downregulated in the catagen profiles include non-membrane bounded organelle, Keratin, cell cycle, and negative regulation of apoptosis, consistent with an inhibition of proliferation, abrogation of differentiation-related gene expression (keratins), loss of hair shaft production (as hair shaft assembly occurs in a non-membrane bound organelle), and increased apoptosis (S7A Fig) that occurs in the transition from anagen to catagen. We also analyzed downregulated functions in the telogen transcriptome profiles. These also include Keratin, non-membrane bounded organelle, and negative regulation of apoptosis, and epithelial development (S7B Fig). Collectively, these findings suggested that the programs driving proliferation, survival, and keratin-mediated hair shaft assembly are silenced in the catagen-to-telogen transition.

**Fig 6:**
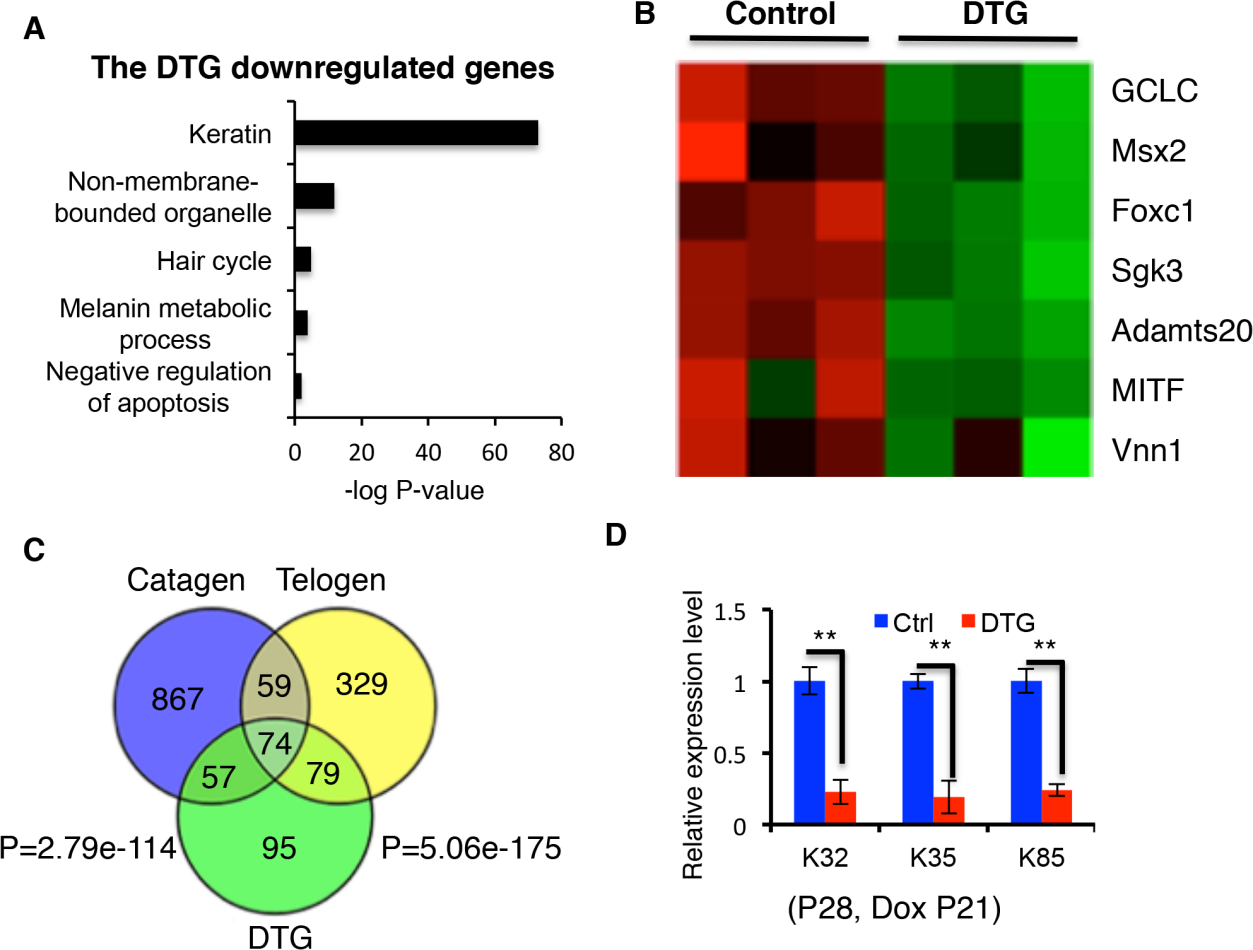
Comparison of downregulated genes in catagen, telogen and DTG transcriptome profiles. (**A**) Gene ontology analysis of downregulated genes in DTG profiles. (**B**) Heatmaps for inhibitors of apoptosis downregulated in DTG profiles. (**C**) Overlap of downregulated genes in the catagen, telogen and DTG profiles. (**D**) Q-PCR for *K32*, *K35*, and *K85* in control and DTG skin at P28, following Dox treatment at P21. ** p < 0.01.

To understand the molecular mechanisms of how *miR-22* contributes to the termination of the hair cycle, we performed transcriptome profiling on control and DTG backskin after 7 days of Dox treatment (P21-P28) (S6 Fig). We identified 305 downregulated genes and 137 upregulated genes with a significance q<0.05 and a fold change >1.5. Enriched downregulated GO clusters upon *miR-22* induction included keratin, non-membrane bounded organelle, hair cycle, melanin metabolism process and negative regulation of apoptosis (Fig 6A). These gene expression patterns are in good agreement with our phenotypic observations of decreased differentiation and increased apoptosis resulting from ectopic induction of *miR-22* (Figs 2–4), and in agreement with functions normally downregulated upon transition from anagen to catagen and telogen, suggesting that *miR-22* may be a critical regulator of this transition.

Anti-apoptotic genes that are suppressed by miR-22 induction include *GCLG*, *Msx2*, *Foxc1*, *Sgk3*, *Adamts20*, *MITF*, and *Vnn1* (Fig 6B) [33–37]. The 305 genes downregulated by miR-22 exhibited considerable overlap with genes downregulated in telogen (153/305, 50.16%) and catagen (131/305, 42.95%) (Fig 6C; S1 Table and S2 Table). Remarkably, among the 153 genes commonly suppressed during telogen and by miR-22 induction were 42 keratin genes (27.45%) (S7C Fig). We confirmed the downregualtion of *K32*, *K35* and *K85* by qRT-PCR (Fig 6D). Interestingly, there are 74 genes commonly downregulated by miR-22 induction, during catagen, and during telogen. These genes include 27 keratin genes and the transcriptional regulators of keratinocyte differentiation *Dlx3*, *Foxn1* and *Msx2* (Fig 6C and S3 Table). These findings suggest that miR-22 directly represses a transcriptional program governing hair shaft assembly and keratinocyte differentiation.

The much smaller set of genes upregulated by miR-22 induction (137) had little in common with the Catagen (5 genes) and Telogen (4 genes) signatures (S8 Fig), further suggesting that the phenotypic consequences of *miR-22* activation are through transcript repression.

### Multiple hair differentiation regulators are *miR-22* direct targets

**Fig 7:**
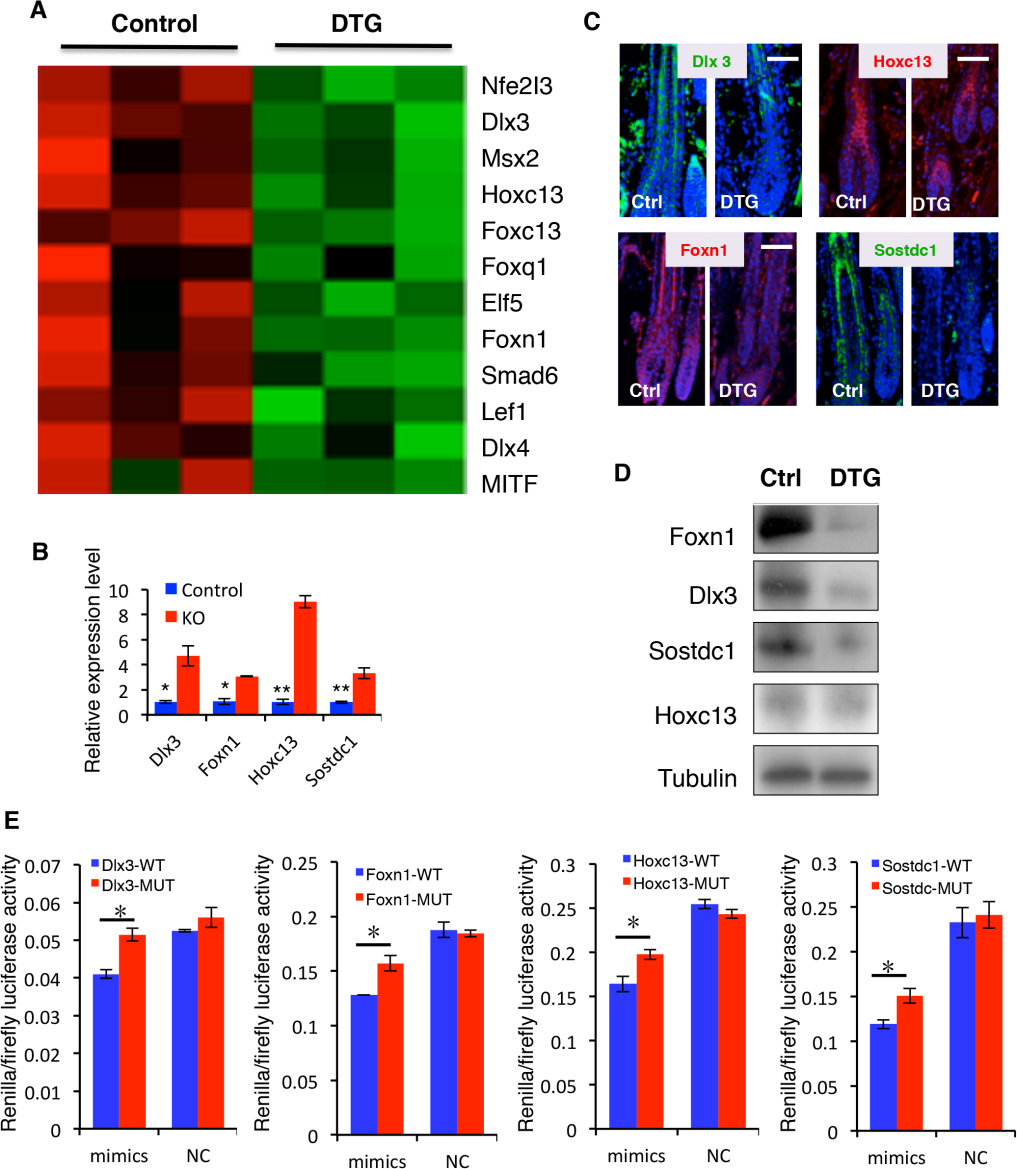
Identification of direct *miR-22* target genes. (**A**) Heatmaps of transcription factors downregulated in DTG skin. (**B**) qPCR for *Foxn1*, *Dlx3*, *Sostdc1* and *Hoxc13* in control and *miR-22* KO skin at P18. * p < 0.05; ** p < 0.01. (**C**) Immunofluorescence for Foxn1, Dlx3, Sostdc1 and Hoxc13 in control and DTG skin. (**D**) Representative immunoblot for Foxn1, Dlx3, Sostdc1 and Hoxc13 proteins in control and DTG skin. Tubulin was used as a control. (**E**) Luciferase reporter activity of *Foxn1*, *Dlx3*, *Sostdc1* and *Hoxc13* wild type and mutant 3’UTR constructs based on 3 independent experiments. ** p < 0.01; * p < 0.05.

To shed light on how *miR-22* negatively regulates keratinocyte phenotypic gene expression, we initially identified all downregulated transcription factors in the DTG transcriptome profiles. Included were *Dlx3*, *Msx2*, *Hoxc13*, *Foxn1*, *Nfe2I3*, *Elf5*, *Smad6*, *Lef1*, *Dlx4* and *MITF*, mostly known regulators of keratinocyte differentiation (Fig 7A). Among them, *Dlx3*, *Hoxc13*, *Foxn1* and *Lef1* are capable of directly controlling keratin gene expression [11] [10,13,15]. Identification of *miR-22* binding sites using TargetScan indicated that 3’UTR of transcription factors *Dlx3*, *Hoxc13*, *Foxn1*, *Nfe2I3* and *Elf5* are direct *miR-22* targets (S9A Fig). The secreted BMP antagonist *Sostdc1*, which promotes migration of DP progenitors [38], also contains *miR-22* binding sites and is significantly downregulated in DTG transcriptome profiles (S9A Fig). In addition, *Foxc1*, *Sgk3*, and *MITF* contain miR-22 binding sites in their 3’UTR (S6B Fig), which are downregulated anti-apoptotic genes [33–35]. We focused on the transcription factors that directly regulate keratin gene expression (*Dlx3*, *Foxn1*, *Hoxc13*) and the BMP antagonist *Sostdc1* for functional validation as *miR-22* targets. Q-PCR results confirmed that *Dlx3*, *Hoxc13*, *Foxn1* and *Sostdc1* mRNA levels were markedly reduced in the DTG skin (S7D Fig), and significantly upregulated in miR-22 KO skin (Fig 7B). Immunofluorescence and Western blotting confirmed that Dlx3, Foxn1 and Sostdc1 protein levels are greatly deceased in DTG follicles, however Hoxc13 protein levels did not appear significantly altered (Fig 7C and 7D). To further test whether 3’UTRs of these genes are direct targets of *miR-22*, we constructed luciferase reporters for the *Dlx3*, *Hoxc13*, *Foxn1* and *Sostdc1* 3’UTRs, as well as reporter constructs in which the predicted *miR-22* binding sites were mutated (S9C Fig). *miR-22* mimics significantly repressed wild type *Dlx3*, *Hoxc13*, *Foxn1* and *Sostdc1* 3’UTR reporter activity, but had no significant influence on the mutant reporters (Fig 7E). These results further demonstrate that *miR-22* directly regulates the *Dlx3*, *Hoxc13*, *Foxn1* and *Sostdc1* 3’UTRs.

Taken together, our findings demonstrate that *miR-22* acts as a pleiotropic regulator of a transcriptional program that regulates hair follicle stem and progenitor cell expansion, migration, and subsequent differentiation and hair shaft formation.

## Discussion

**Fig 8:**
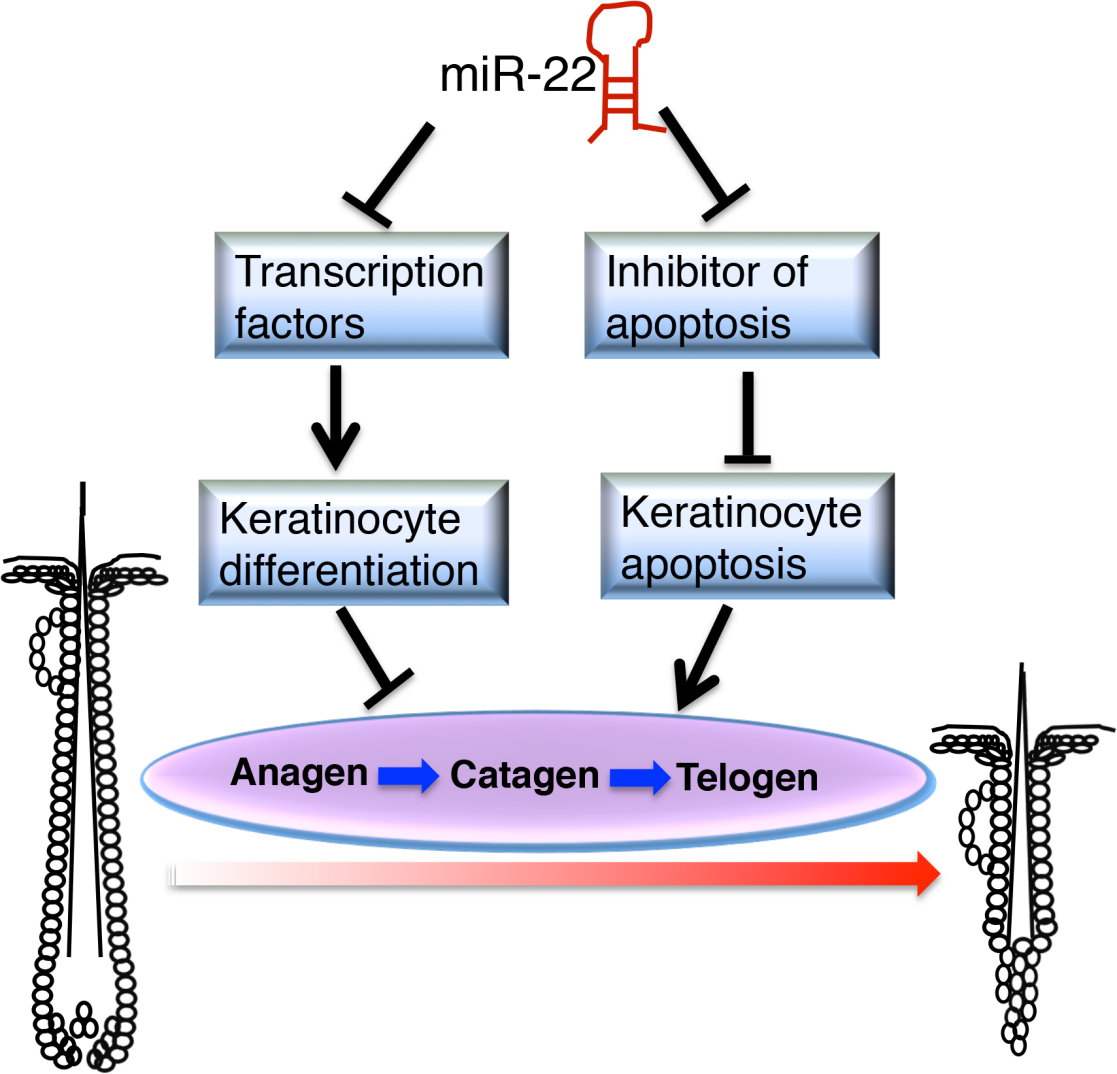
Schematic of *miR-22* working model in hair cycling.

In this study we demonstrate that *miR-22* promotes hair follicle involution through negative post-transcriptional regulation of keratinocyte progenitor expansion, differentiation and hair shaft assembly in the catagen and telogen phases. The fact that *miR-22* overexpression promotes anagen-to-catagen transition and maintains hair follicles in telogen, coupled with the observed delayed entry to catagen from anagen and accelerated transition from telogen to anagen upon deletion of *miR-22*, point to an important role of *miR-22* in controlling termination of the hair cycle. *MiR-22* appears to promote the anagen-to-catagen transition through increasing apoptosis and repressing master regulators of keratinocyte differentiation (Fig 8).

During hair cycling, the HF undergoes dynamic changes in morphology [39]. In the anagen phase, the TA matrix cells undergo proliferation and differentiation to form the hair shaft. The hair shaft is a highly keratinized product of the hair follicle, formed by type I and type II keratins, which generate intermediate filaments in the cytoplasm and contribute to cytoskeleton formation [39]. The transition from anagen, to catagen, and to telogen is a physiological involution, with HF rapidly degenerating and shortening [7]. GO analysis of catagen and telogen transcriptome profiles revealed that *keratin* genes are continuously downregulated from catagen to telogen phases, supporting the idea that the involution requires the repression of keratinocyte differentiation and hair shaft assembly [16]. Until now, the molecular regulators terminating hair shaft generation and promoting follicle involution were largely unknown. In the current study we identify *miR-22* as a critical post-transcriptional regulator repressing keratinocyte differentiation and promoting transition from anagen to catagen and maintaining telogen phases.

*Mir-22* represses a number of transcription factors that promote keratinocyte differentiation and hair shaft formation, including *Dlx3*, *Msx2*, *Hoxc13*, *Foxn1*, *Nfe2I3*, *Elf5*, *Smad6*, *Lef1*, *Dlx4* and *MITF* in the DTG skin. Further, we identified *miR-22* binding sites in the 3’UTR of *Nfe2I3*, *Elf5*, *Dlx3*, *Hoxc13* and *Foxn1*. Our results demonstrated that *miR-22* directly regulates the expression of *Dlx3*, *Hoxc13* and *Foxn1* using luciferase activity assay (Fig 7F). Thus, *miR-22* may directly repress these transcription factors to control keratin gene expression and keratinocyte differentiation. Among them, a core of transcription factors including *Dlx3*, *Foxn1*, *Hoxc13* and *Lef1* can directly bind to and activate the promoters of hair keratin genes including *K32*, *K35*, and *K37* [10,11,13]. Consistent with our model, the *miR-22* target *Dlx3* is expressed at its highest levels in anagen, with expression decreasing in catagen and terminating in telogen [11]. Thus, the *Dlx3* expression pattern is inversely correlated to that of *miR-22*, supporting this notion that *miR-22* repression of *Dlx3* contributes to HF involution. Consistent with this notion, deletion of *Dlx3* leads to hair loss by directly repressing hair keratin gene expression [11]. *Dlx3* can directly bind to the promoter of *K32*, *K35* and *Hoxc13* to activate gene expression [11]. The *Dlx3* KO phenotype resembles the *miR-22* overexpression mouse phenotype, indicating that *Dlx3* is a functionally important target of *miR-22* during hair cycling. *Foxn1* is another direct target of *miR-22*. *Foxn1* knockout mice were initially utilized as a nude mouse model [40]. The nude phenotype is predominantly due to the impaired hair shaft formation caused by reduction of hair keratin gene expression [13]. Like Dlx3, Foxn1 is capable of directly regulating keratin gene expression as a transcription factor or it can act indirectly as a co-activator [13]. Another phenotype of *Foxn1* KO mice is defective differentiation of thymic epithelia [41]. Consistently, we found that thymic development is impaired in the *miR-22* overexpressing DTG transgenic mice (S10 Fig), providing further support that *Foxn1* is a functionally important target of *miR-22*. Consistent with the downregulation of keratinocyte differentiation-related transcription factors, 58 keratin genes are markedly reduced in the *miR-22* overexpressing mice. Thus, *miR-22* plays an important role in hair follicle keratinocyte differentiation during hair cycling.

Global deletion of microRNA processing enzymes *Dicer1* and *Drosha* during anagen lead to failed catagen entry and impaired follicle degradation, revealing an essential function of microRNAs in HF regression [25]. Loss of apoptosis might account for this failed follicle degradation, however *Dicer1* and *Drosha* mutant hair matrix cells exhibit a slight increase in apoptosis, suggesting that apoptosis-independent hair regression mechanisms are defective in *Dicer1* and *Drosha* mutant follicles. In the *miR-22* KO mice, we observed elongated anagen follicles and delayed entry into catagen; phenotypes consistent to with the *Dicer1* and *Drosha*-null follicles. Thus, *miR-22* mediated repression of keratinocyte differentiation may partially account for the *Dicer1* and *Drosha* mutant phenotype. However, the *miR-22* KO phenotype is less severe than that observed *Dicer1* or *Drosha* KO mice, suggesting that other microRNAs contribute to HF regression from anagen, to catagen, and then to telogen. Numerous reports have documented the functions of specific microRNAs on hair development [24,27–29]. Among them, *miR-24* and *miR-125b* have expression patterns similar to that of *miR-22* during hair cycling [24]: sharp upregulation at catagen and maximal expression at telogen phase. Consistent with *miR-22*, ectopic expression of *miR-24* in the ORS of hair follicles resulted in hair loss with severe defects in hair follicle morphogenesis. *miR-24* repressed hair keratinocyte differentiation by negatively regulating TCF3, an important upstream regulator of hair differentiation [14,27]. *miR-125b* is another well-characterized microRNA in hair development. Similarly to *miR-22*, *miR-125b* overexpression leads to hair loss by repressing hair differentiation. *miR-125b* directly represses the vitamin D receptor, which plays an important role in hair differentiation [28]. Thus, *miR-22*, *miR-24* and *miR-125b* might synergistically function to promote hair regression by repressing a keratinocyte differentiation regulatory program in the transition into catagen and telogen. This phenomenon may in fact be more broad. In addition to *miR-24*, *miR-125b*, numerous microRNAs including *miR-29a*, *miR-27a*, *miR-27b*, *miR-30a*, *miR-152* and *miR-143* also exhibit the same expression pattern as *miR-22* [24]. Thus, future studies are warranted to determine the combinatorial effects of this suite of microRNAs in hair cycle transitions.

Another important finding in the current study is that miR-22 inhibits hair follicle stem cell colony formation capacity and hair neogenesis. Interestingly, the effects on HFSCs were also observed for *miR-24* and *miR-125b*. *MiR-24* overexpression leads to depletion of HFSCs [27], suggesting a role of *miR-24* in promoting HFSC commitment and loss of self-renewal. Thus, while these microRNAs are important in regulating HFSC self-renewal, they likely have unique functions.

It is well known that catagen is an apoptosis-driven physiological involution. A variety of signaling pathways inducing apoptosis have been identified in the process of hair regression during catagen [16]. These regulators include *c-Kit*, *Bcl-2*, *hairless*, *survivin*, *Bax*, *p53*, *Dkk1*, and transforming growth factors, *TGF-1* and *TGF-2* [16]. In this study we show that *miR-22* promote apoptosis in hair follicles. Several negative regulators of apoptosis were downregulated in the *miR-22* overexpressing mice including *ADAMTS20*, *Foxc1*, *GCLC*, *MITF*, *SGK3*, *Msx2*, *Vnn1*. Among them, *Foxc1*, *MITF*, and *SGK3* contain *miR-22* binding sites. *miR-22* might directly repress these transcripts to activate apoptotic signaling pathways. Thus, our findings suggest that a post-transcriptional mechanism regulating apoptosis might be involved in hair regression.

Interestingly, it has recently been reported that *miR-22* is strongly induced in response to testosterone treatment [42,43]. Male pattern baldness is due to the premature transition from anagen-to-catagen induced by androgens [3]. Thus, this raises the hypothesis that *miR-22* might function in the pathogenesis of Androgenic alopecia (AGA).

## Materials and Methods

### Ethics statement

This study was performed in strict accordance with the recommendations in the Regulations of Beijing Laboratory Animal Management. The protocol was approved by the Institutional Animal Care and Use Committee of China Agricultural University (approval number: SKLAB-2011-04-03).

### Mice

To generate *TRE-miR-22* mice, the *mmu-miR-22* coding sequence was amplified by PCR from mouse genomic DNA (Forward Primer: 5’- GTGGATCCGCTAGA GCCAGGTTTG- 3’ and Reverse primer: 5’- GCTCTAGAATTTCTTCCCACTGCC -3’), cloned into *Bam*H I/*Xba*I sites of the pTRE2 plasmid (Clontech) to generate *pTRE-miR-22* construct. The *TRE-miR-22* transgenic mice were generated using standard protocols. *K14-rtTA* transgenic mice (#007678) and *miR-22* ^-/-^ mice (#018155) were purchased from the Jackson Laboratory and backcrossed into the FVB strain.

### Microarray and data analysis

For mRNA microarrays, total RNA samples of dorsal skin were extracted from P28 DTG and control mice (Dox since P21) using TRIzol reagent (Invitrogen). Then the RNA samples were submitted to the Beijing CapitalBio Corporation and analyzed on Affymetrix mouse 430_2 arrays. The data were analyzed using Significant Analysis of Microarray (SAM) software to identify the differentially expressed genes. Differentially expressed transcripts were defined by a fold-change (FC) ≥1.5 and a *q*-value <5%. The transcriptome profiles of catagen and telogen are published in [32]. The differentially expressed genes were identified with a significance P value < 0.05 and an FC of 1.5. We utilized DAVID Functional Annotation Bioinformatics Microarray Analysis software to analyze gene ontology (GO) based on the KEGG pathway database.

### Histology, immunochemistry and immunofluorescence

Immunochemistry and immunofluorescence were performed as described previously [44]. Skin samples were fixed in 4% PFA, paraffin-embedded 5-μm sections were stained with hematoxylin and eosin (H&E). For immunofluorescence staining, paraffin sections were microwave pretreated, and incubated with primary antibodies, then incubated with secondary antibodies (Invitrogen) and counterstained with DAPI in mounting media. The following antibodies and dilutions were used: K14 (rabbit, 1:1000, Covance), K14 (mouse, 1:500, Abcam),K5 (rabbit, 1:1000, Covance), Sox9 (rabbit, 1:100, Santa Cruz), AE13(mouse, 1:100, Abcam), AE15 (mouse, 1:100, Abcam), BrdU (rat, 1:200, Abcam), Cleaved Caspase-3 (rabbit, 1:100, Cell Signaling), Gata-3 (mouse, 1:100, Santa Cruz), K17 (rabbit, 1:100, Abcam), Foxn1 (mouse, 1:100, Santa Cruz), Dlx3 (rabbit, 1:100, Santa Cruz), Sostdc1 (rabbit, 1:100, Abcam), Hoxc13 (mouse, 1:100, Sigma), Lef1 (rabbit, 1:100, Cell Signaling), Ki67 (mouse, 1:100,Novacastra), Bmpr1a (rabbit, 1:100, Abcam), Msx2 (rabbit, 1:200, Santa Cruz), k15 (mouse, 1:150, Vector Labs), CD49f (rat,1:100,BD), p63 (mouse, 1:200, Santa Cruz), K10 (rabbit, 1:1000, Covance), Loricrin (rabbit, 1:500, Covance), K1 (rabbit, 1:500, Covance).

### Wounding and whole-mount hair follicle neogenesis assay

The wounding and wound-induced hair neogenesis analyses were performed as described [45]. The mice were anaesthetized with ketamine/xylazine. A 1 cm^2^ full thickness excision of skin was made on the mid back of 3 week-old mice. To detect newly developing hair follicles in the wound, we incubated the skin in 20 mM EDTA in PBS at 37°C overnight. Then, we gently peeled the epidermal portion off under a dissecting microscope, fixed the epidermis in 4% PFA for 1 h, rinsed with PBS, blocked with 3% H_2_O_2_, and performed immunostaining for KRT17 in 1.5 ml eppendorf tubes. We fixed the dermis in acetone at 4°C overnight, rinsed it with PBS, and then incubated with NBT/BCIP (Roche) for 30 min at 37°C. We stopped the reaction with 20 mM EDTA in PBS.

### FACS

Subcutaneous fat was removed by a scalpel and the whole skin was placed dermis side down in Trypsin (GIBCO) at 4°C O/N. Single-cell suspensions were obtained by scraping the skin to remove the epidermis and HFs from the dermis. The cells were washed with PBS containing 5% of fetal bovine serum (FBS), then filtered through 70 μm cell strainers, followed by 40 μm. Cell suspensions were incubated with the appropriate antibodies for 90 min on ice. The following antibodies were used: Alpha6-PE (1:500, eBioscience), CD34-eFluor660 (1:100, eBioscience). DAPI was used to exclude dead cells. Cell isolations were performed on MoFlo XDP sorters equipped with Summit 5.2 software (Beckman Coulter).

### Colony-forming assay

The colony-forming assay was performed according to previously reported method [46]. 3,000 viable cells per well were plated onto mitomycin-treated 3T3 fibroblasts in E-media supplemented with 15% serum and 0.3 mM calcium using a six-well plate. After 14 days in vitro, to visualize colony number and morphology, cells were stained with 1% Rhodamine B (Sigma). The growth medium was changed every second day. To induce terminal differentiation, serum was reduced to 5%, and calcium was raised to 1.5 mM.

### *miR-22 in situ* hybridizations

*miR-22 in situ* hybridizations were performed based on the previously described protocol [47]. Double DIG-labeled miR-22 and scrambled LNA probes (Exiqon) were hybridized at 55°C. *In situ* signals were detected by staining with Anti-Digoxigenin-AP antibody (Roche) and developing using BM purple substrate (Roche).

### Quantitative PCR

Total RNA was isolated from dorsal skin using the mirVanaTM RNA Isolation kit following manufacturer’s instructions (Ambion). Each RNA sample was reverse transcribed with the M-MLV Reverse Transcriptase (Sigma) using Oligo (dT) primers. Real-time PCR was performed with the LightCycler 480 SYBR Green I master mix on a LightCycler 480 real-time PCR system (Roche). For microRNA expression, mature miR-22 was quantified using TaqMan microRNA assays according to the manufacturer’s instructions. U6 snRNA was used as an internal control (Applied Biosystems). Q-PCR primers were listed in S4 Table.

### Dual luciferase activity assays

To generate reporter constructs for luciferase assays, segments of about 650 bp in length containing predicted miRNA target sites in the 3’UTRs of *Dlx3*, *Sostdc1*,*Hoxc13* and *Foxn1* were cloned into the psiCHECK-2 vector (Promega) between the XhoI or SgfI and NotI sites immediately downstream of the *Renilla* luciferase gene. To generate reporters with mutant 3’UTRs, six nucleotides (GCAGCT) in the target site complementary to the position 2–7 of the *Mir22* seed region were mutated to TAGATC by a QuikChange Site-Directed Mutagenesis kit according to the manufacturer’s protocol (Stratagene).

293T cells were seeded in 96-well plates one day before transfection. Ten nanograms of each reporter construct was co-transfected with miR-22 mimic or a negative control at a final concentration of 50 nM into 293T cells using Lipofectamine 2000 according to the manufacturer’s protocol (Invitrogen). After 24h, firefly and *Renilla* luciferase activities were measured with the Dual-Glo luciferase assay system according to the manufacturer’s instructions (Promega).

The primers used for amplifying 3’-UTR of candidate target genes of miR-22 were listed in S4 Table.

### Western blot

P24 Dorsal skin (Dox since P21) was homogenized in ice-cold lysis buffer (Beyotime, China) with 1% PMSF (Phenylmethylsulfonyl fluoride). Samples were centrifuged at 10,000g for 20 min at 4℃ and the protein concentration of the resulting supernatant was determined by the BCA (bicinchoninic acid) protein assay kit (Beyotime, China). Proteins (30μg) were separated by SDS-PAGE electrophoresis and subsequently transferred to PVDF membranes. Membranes were blocked with 5% nonfat dry milk in incubation buffer and incubated with primary antibodies against Tubulin, Dlx3, Sostdc1, Hoxc13 and Foxn1. Bound antibody was detected with peroxidase-linked secondary antibody and a chemiluminescence detection system.

## Supporting Information

S1 Fig *miR-22* expression during hair cycling. (**A**) qPCR analysis of *miR-22* in mouse backskin at P9 (anagen), P17 (catagen) and P21 (telogen). ** p < 0.01. (**B**) *In situ* hybridization for *miR-22* in mouse backskin at P30. Scramble was used as a negative control. The DTG backskin was used as a positive control. 100 m. (**C**) The higher magnification image was indicated by the dashed box in B. Arrows point to *in situ* signal at the out root sheath of hair follicle. Arrowheads point to *in situ* signal at the cells of hair matrix in close to the Dermal papilla. Scale bar: 25 μm. (**D**) qPCR analysis for *miR-22* on 2-month-old and 18-month-old mouse backskin. ** p < 0.01.
(TIF)

S2 Fig *miR-22* promotes the transition from anagen to catagen and retards the transition from telogen to anagen. (**A**) The DTG pups exhibit thinning hair coat at P12 when they are treated with Dox at embryonic day (E) 15. The histology of control and DTG pups. (**B**) External hair regrowth is delayed in the DTG mice after depilation. Both control and DTG mice were treated with Dox at P53. Hairs were plucked at P56. Photographed at P65. (DTG n = 3; control n = 3). (**C**) Representative histological images of dorsal skin from Dox-treated DTG mice and their Dox-treated WT or *K14-rtTA* (control) littermates at the indicated postnatal ages (P). Both control and DTG mice were treated with Dox at P1. Each time point, DTG n = 3; Control, n = 3. Scale bar, 100 m. (**D**) Higher magnification images of Fig 3A indicated by dashed box. Arrows point to hair bulb. Scale bar: 100 m. (**E**) qPCR for MMP11, K16, TGF2, SPINK12 in Control and DTG skin at P16, following Dox treatment at P1. (**F**) Higher magnification images of Fig 3D indicated by dashed box. Arrows point to hair bulb. (**G**) Quantification of hair follicle length in 3 control and 3 KO mice at P9 and P26 in Fig 3D. Percentage of long hair follicles in 3 control and 3 KO mice at P21 in Fig 3D. ** p < 0.01; * p < 0.05.
(TIF)

S3 Fig *miR-22* induction promotes cell proliferation of epidermis. (**A**) HE staining, Ki67 and p63 immunofluorescence for Control and DTG skin at P30, following Dox treatment at P1. Scale bar: 50 μm. (**B**) Quantification of Control and DTG epidermal thickness. ** p < 0.01. (**C**) Immunofluorescence for Loricrin, K1/K14 and p63/K10 in Control and DTG skin at P30, following Dox treatment at P1. (**D**) Quantification of Ki67 and p63 positive cells in Control and DTG skin. * p < 0.05; ** p < 0.01.
(TIF)

S4 Fig *miR-22* induction represses cell differentiation and promotes apoptosis. (**A**) Schematic of BrdU experiment under hair depilation condition. Immunofluorescence for BrdU in control and DTG skin at 11 hours post BrdU pulse. Scale bar, 50 m. Quantification of BrdU^+^ cells per Matrix and per precotex in the control and DTG hair follicles at 11 hours post BrdU pulse. (**B**) Quantification of Lef1^+^ cells in 3 control and 3 miR-22 DTG mice in Fig 4C. Quantification of Lef1^+^, Gata3^+^ and Casp3^+^ cells in 3 control and 3 KO mice in Fig 4C, 4D and 4G. ** p < 0.01; * p < 0.05.
(TIF)

S5 Fig *miR-22* does not affect the number of hair follicle stem cells. (**A**) Immunofluorescence for Sox9 in control and DTG hair follicles. Scale bar: 50 m. (**B**) FACS profiles of CD34-eFluor660 and CD49f-PE in hair cell suspensions from Control and DTG mice. (**C**) Representative images of 1 cm^2^ skin wounding in control and DTG mice at P21.
(TIF)

S6 Fig Heatmap and hierarchical clustering of transcriptome profiles of 3 Control and 3 DTG mice treated with Dox for 1 week from P21 to P28. (TIF)

S7 Fig Analysis of downregulated genes in catagen, telogen and DTG transcriptome profiles. (**A**) Gene ontology analysis of downregulated genes in the catagen profiles. (**B**) Gene ontology analysis of downregulated genes in the telogen profiles. (**C**) List of common downregulated keratin genes in telogen and DTG profiles. (**D**) qPCR for *Foxn1*, *Dlx3*, *Sostdc1* and *Hoxc13* in control and DTG skin. ** p < 0.01.
(TIF)

S8 Fig Comparison of upregulated genes in catagen, telogen and-the DTG transcriptome. (**A**) Gene ontology analysis of upregulated genes in catagen. (**B**) Gene ontology analysis of upregulated genes in telogen. (**C**) Gene ontology analysis of upregulated genes in DTG skin. (**D**) Overlap of upregulated genes in catagen and DTG skin. (**E**) Overlap of downregulated genes in telogen and DTG skin.
(TIF)

S9 Fig Identification of miR-22 binding sites in transcription factors and negative regulators of apoptosis. (**A**) The downregulated transcription factors containing *miR-22* binding sites. (**B**) The downregulated negative regulators of apoptosis containing *miR-22* binding sites. (**C**) Mutant binding sites of genes, *Dlx3*, *Foxn1*, *Hoxc13* and *Sostdc1*.
(TIF)

S10 Fig Thymus development is defective in the Dox treated DTG mice. Gross pictures of thymus in the Control and DTG mice treated with Dox from P1 to P49. Arrows point to thymus.
(TIF)

S1 Table List of 131 commonly downregulated genes between DTG and Catagen. (PDF)

S2 Table List of 153 commonly downregulated genes between DTG and Telogen. (PDF)

S3 Table List of 74 commonly downregulated genes among DTG, Catagen and Telogen. (PDF)

S4 Table The sequences of primer used in this paper. (PDF)
