# Supplementary figures and images for: Post-transcriptional Regulation of Keratinocyte Progenitor Cell Expansion, Differentiation and Hair Follicle Regression by miR-22
Source: PLoS Genet. 2015 May 28;11(5):e1005253. doi: 10.1371/journal.pgen.1005253 (PMC4447420; doi:10.1371/journal.pgen.1005253)

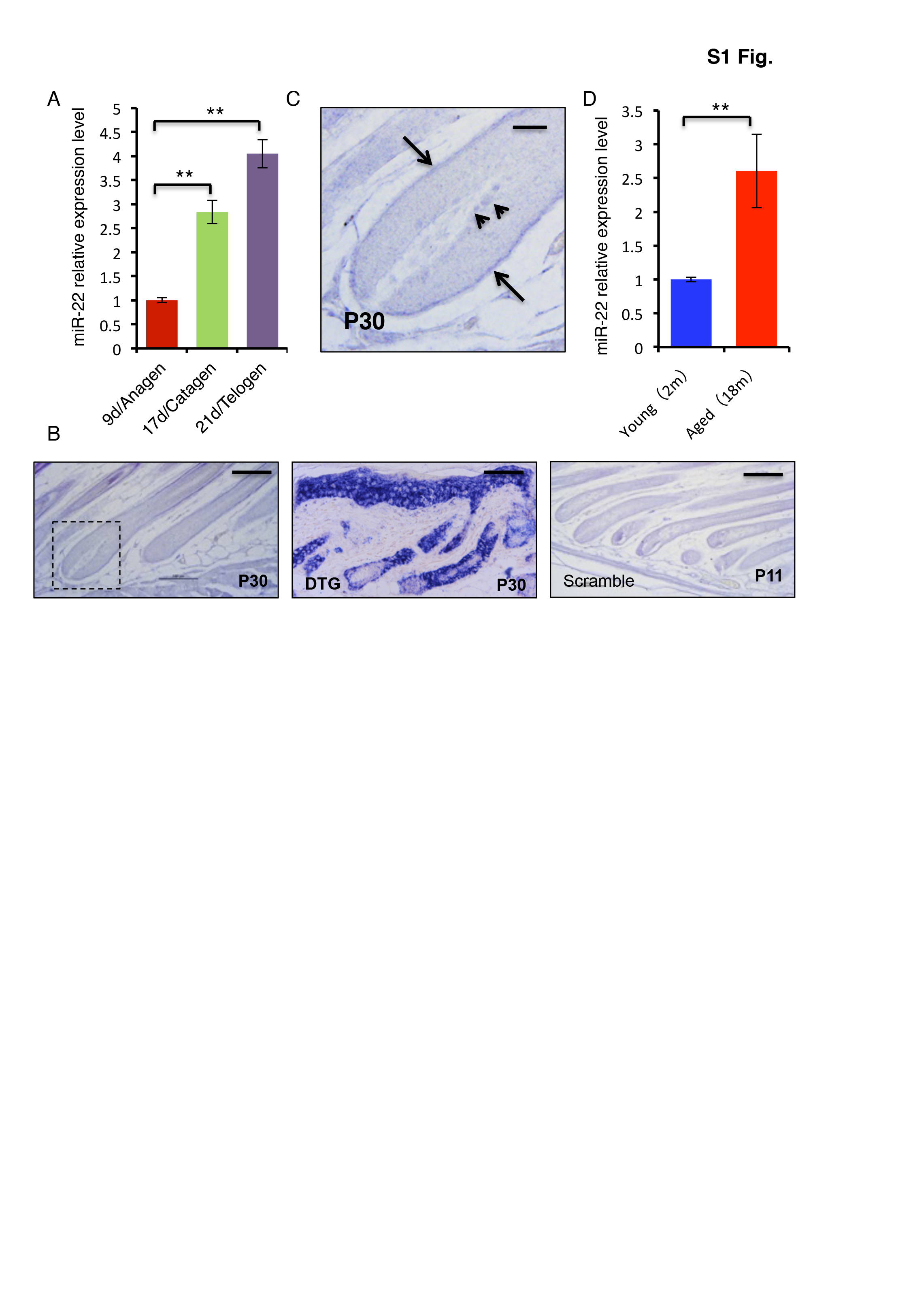

Supplement: S1 Fig — (A) qPCR analysis of miR-22 in mouse backskin at P9 (anagen), P17 (catagen) and P21 (telogen). ** p < 0.01. (B) In situ hybridization for miR-22 in mouse backskin at P30. Scramble was used as a negative control. The DTG backskin was used as a positive control. 100 m. (C) The higher magnification image was indicated by the dashed box in B. Arrows point to in situ signal at the out root sheath of hair follicle. Arrowheads point to in situ signal at the cells of hair matrix in close to the Dermal papilla. Scale bar: 25 μm. (D) qPCR analysis for miR-22 on 2-month-old and 18-month-old mouse backskin. ** p < 0.01. (TIF) [file pgen.1005253.s001.tif]

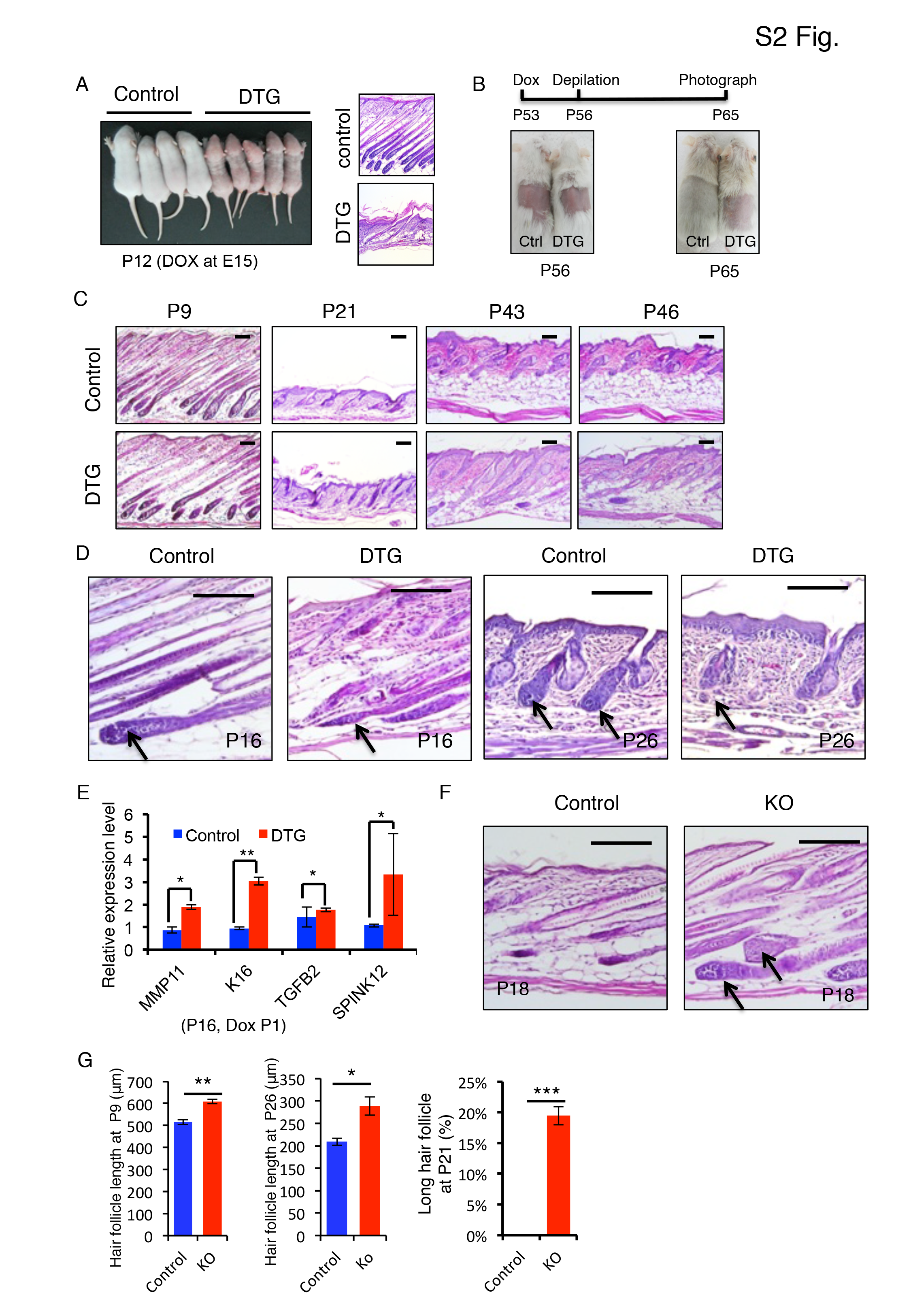

Supplement: S2 Fig — (A) The DTG pups exhibit thinning hair coat at P12 when they are treated with Dox at embryonic day (E) 15. The histology of control and DTG pups. (B) External hair regrowth is delayed in the DTG mice after depilation. Both control and DTG mice were treated with Dox at P53. Hairs were plucked at P56. Photographed at P65. (DTG n = 3; control n = 3). (C) Representative histological images of dorsal skin from Dox-treated DTG mice and their Dox-treated WT or K14-rtTA (control) littermates at the indicated postnatal ages (P). Both control and DTG mice were treated with Dox at P1. Each time point, DTG n = 3; Control, n = 3. Scale bar, 100 m. (D) Higher magnification images of Fig 3A indicated by dashed box. Arrows point to hair bulb. Scale bar: 100 m. (E) qPCR for MMP11, K16, TGF2, SPINK12 in Control and DTG skin at P16, following Dox treatment at P1. (F) Higher magnification images of Fig 3D indicated by dashed box. Arrows point to hair bulb. (G) Quantification of hair follicle length in 3 control and 3 KO mice at P9 and P26 in Fig 3D. Percentage of long hair follicles in 3 control and 3 KO mice at P21 in Fig 3D. ** p < 0.01; * p < 0.05. (TIF) [file pgen.1005253.s002.tif]

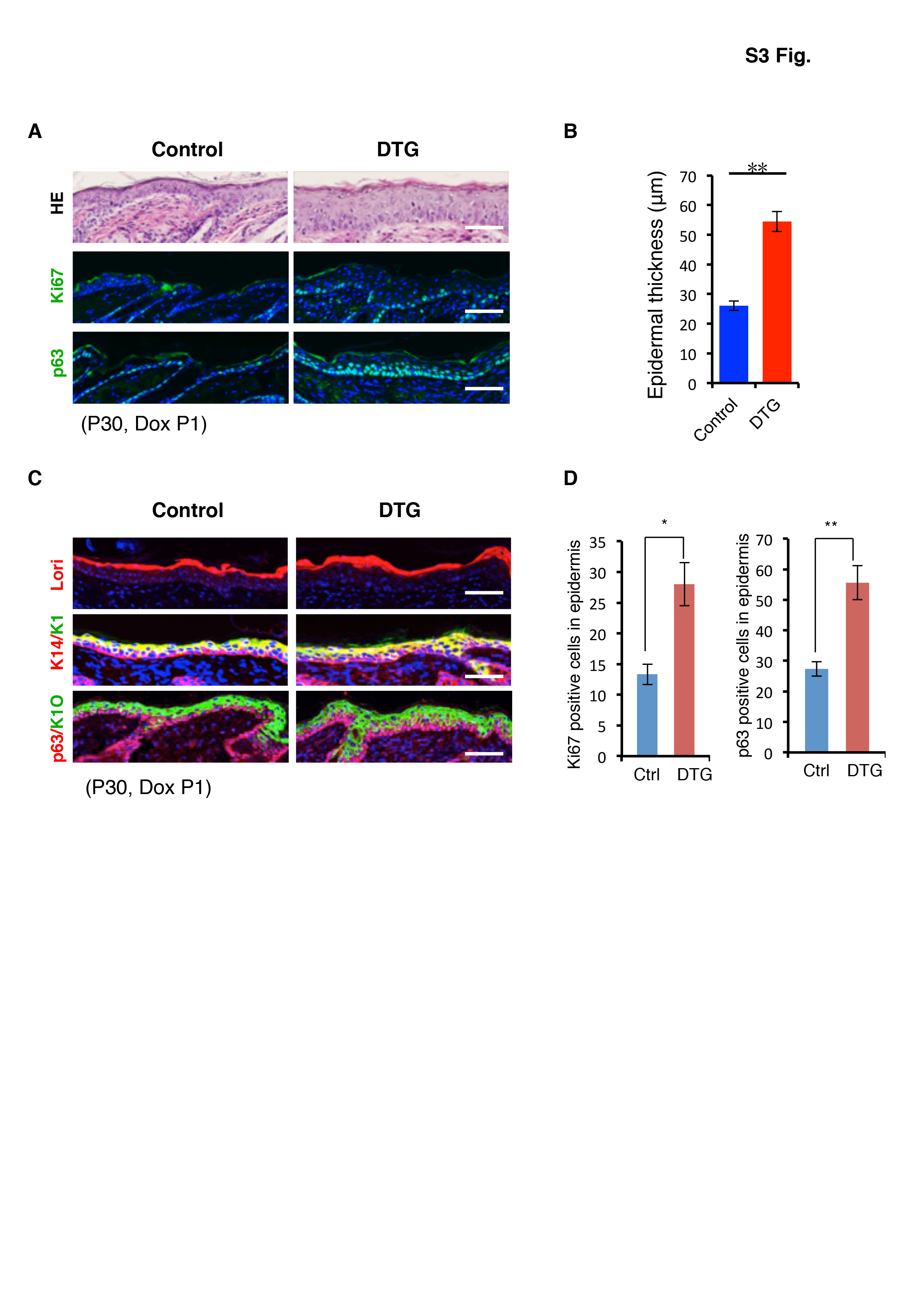

Supplement: S3 Fig — (A) HE staining, Ki67 and p63 immunofluorescence for Control and DTG skin at P30, following Dox treatment at P1. Scale bar: 50 μm. (B) Quantification of Control and DTG epidermal thickness. ** p < 0.01. (C) Immunofluorescence for Loricrin, K1/K14 and p63/K10 in Control and DTG skin at P30, following Dox treatment at P1. (D) Quantification of Ki67 and p63 positive cells in Control and DTG skin. * p < 0.05; ** p < 0.01. (TIF) [file pgen.1005253.s003.tif]

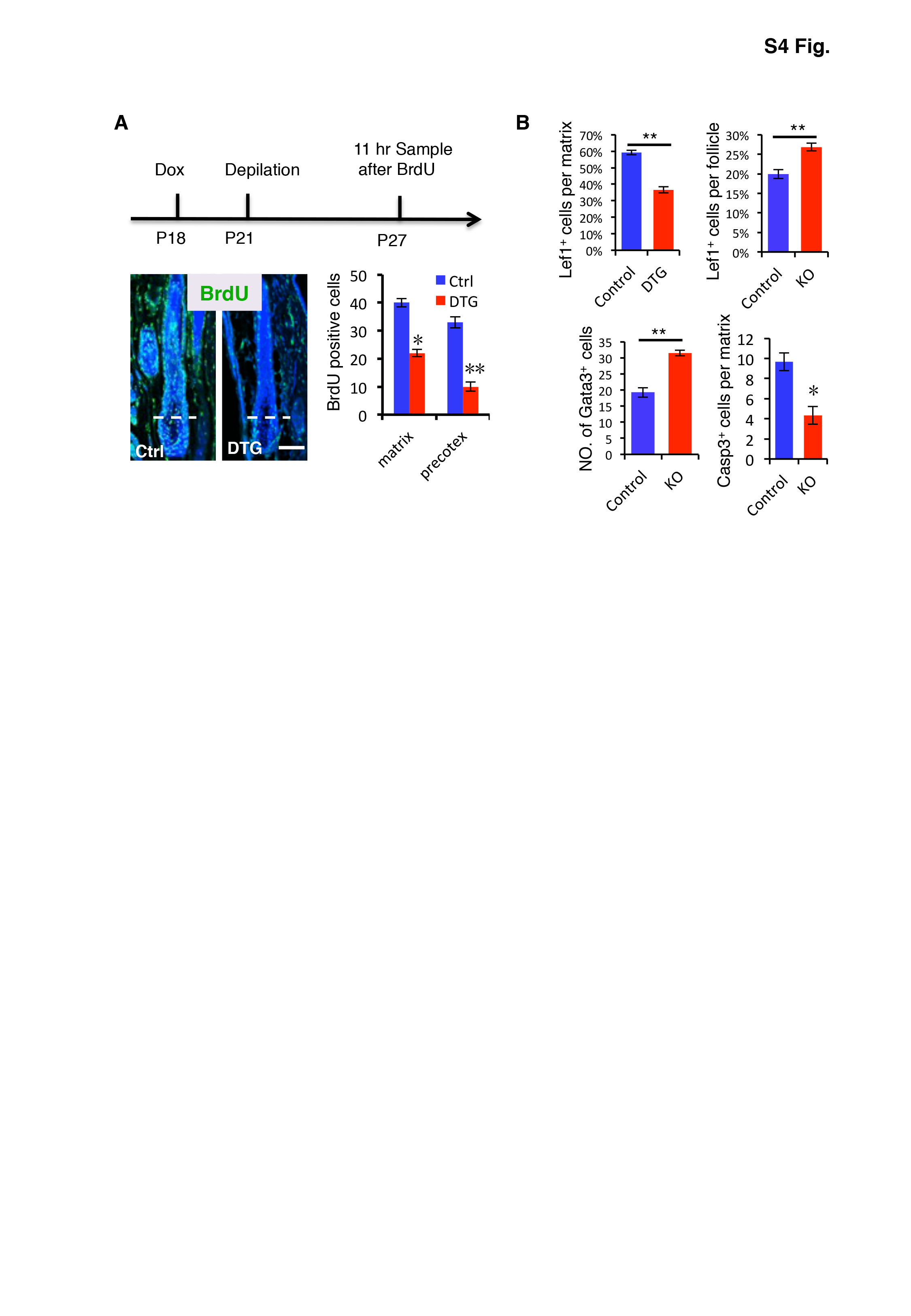

Supplement: S4 Fig — (A) Schematic of BrdU experiment under hair depilation condition. Immunofluorescence for BrdU in control and DTG skin at 11 hours post BrdU pulse. Scale bar, 50 m. Quantification of BrdU+ cells per Matrix and per precotex in the control and DTG hair follicles at 11 hours post BrdU pulse. (B) Quantification of Lef1+ cells in 3 control and 3 miR-22 DTG mice in Fig 4C. Quantification of Lef1+, Gata3+ and Casp3+ cells in 3 control and 3 KO mice in Fig 4C, 4D and 4G. ** p < 0.01; * p < 0.05. (TIF) [file pgen.1005253.s004.tif]

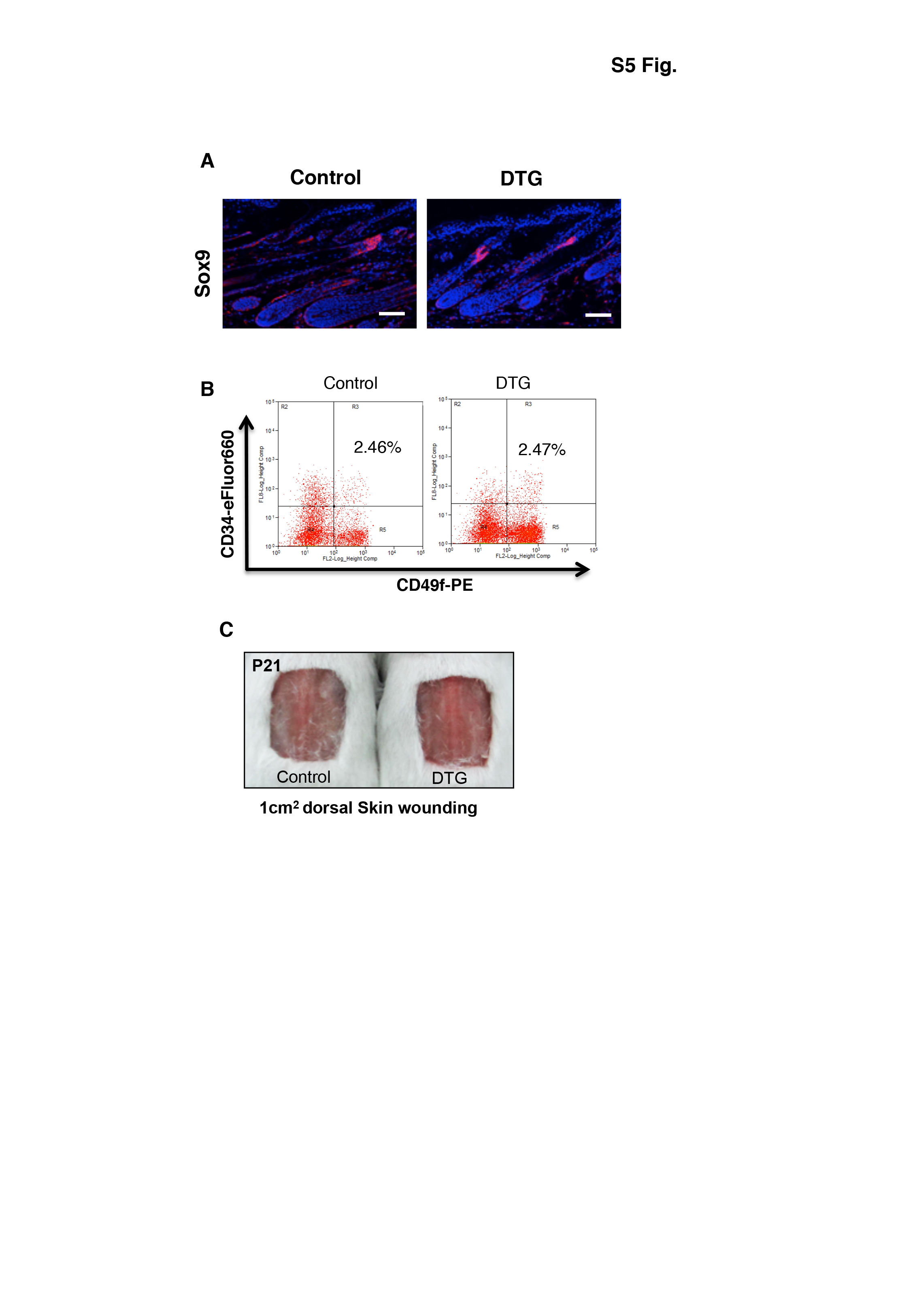

Supplement: S5 Fig — (A) Immunofluorescence for Sox9 in control and DTG hair follicles. Scale bar: 50 m. (B) FACS profiles of CD34-eFluor660 and CD49f-PE in hair cell suspensions from Control and DTG mice. (C) Representative images of 1 cm2 skin wounding in control and DTG mice at P21. (TIF) [file pgen.1005253.s005.tif]

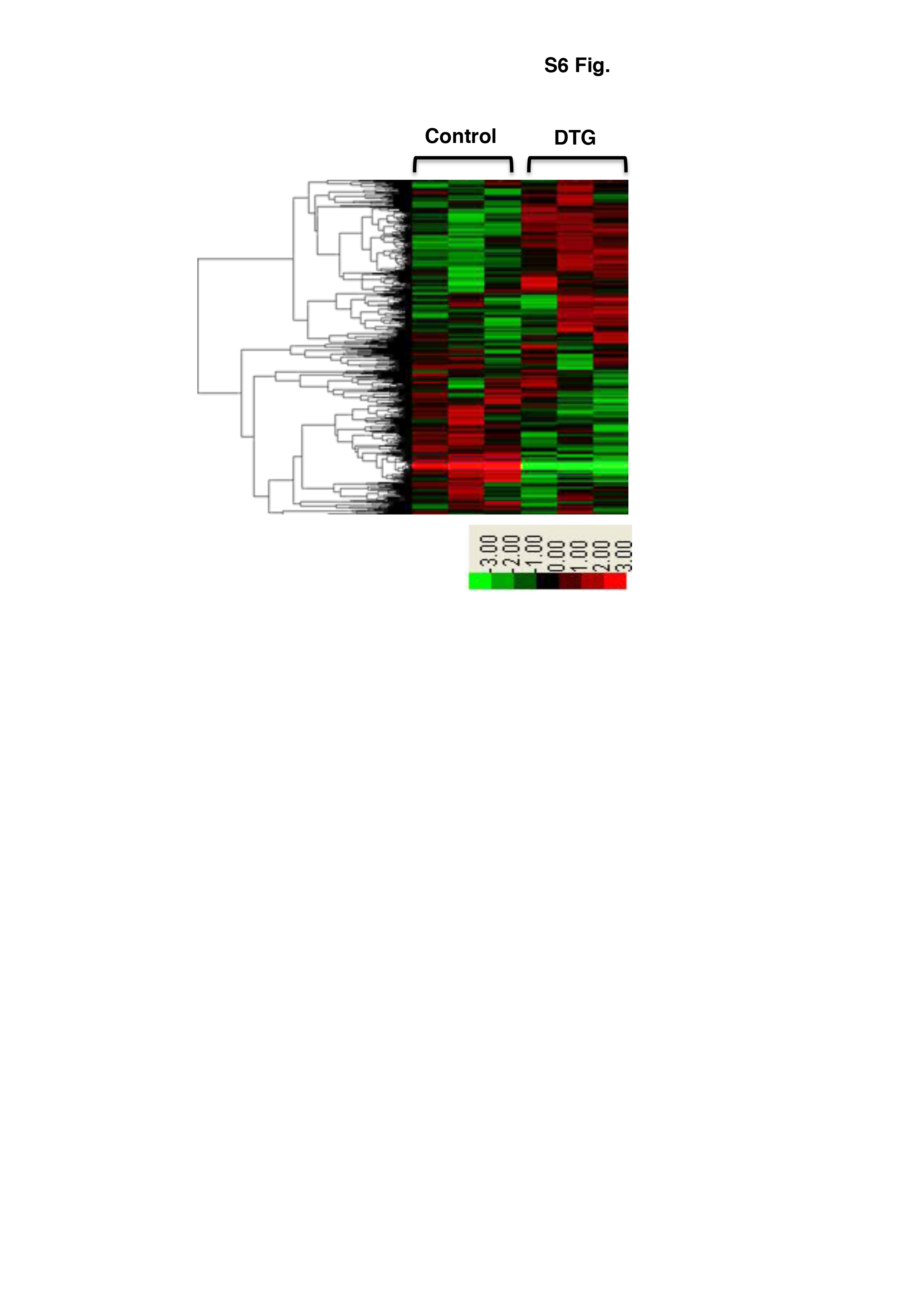

Supplement: S6 Fig — (TIF) [file pgen.1005253.s006.tif]

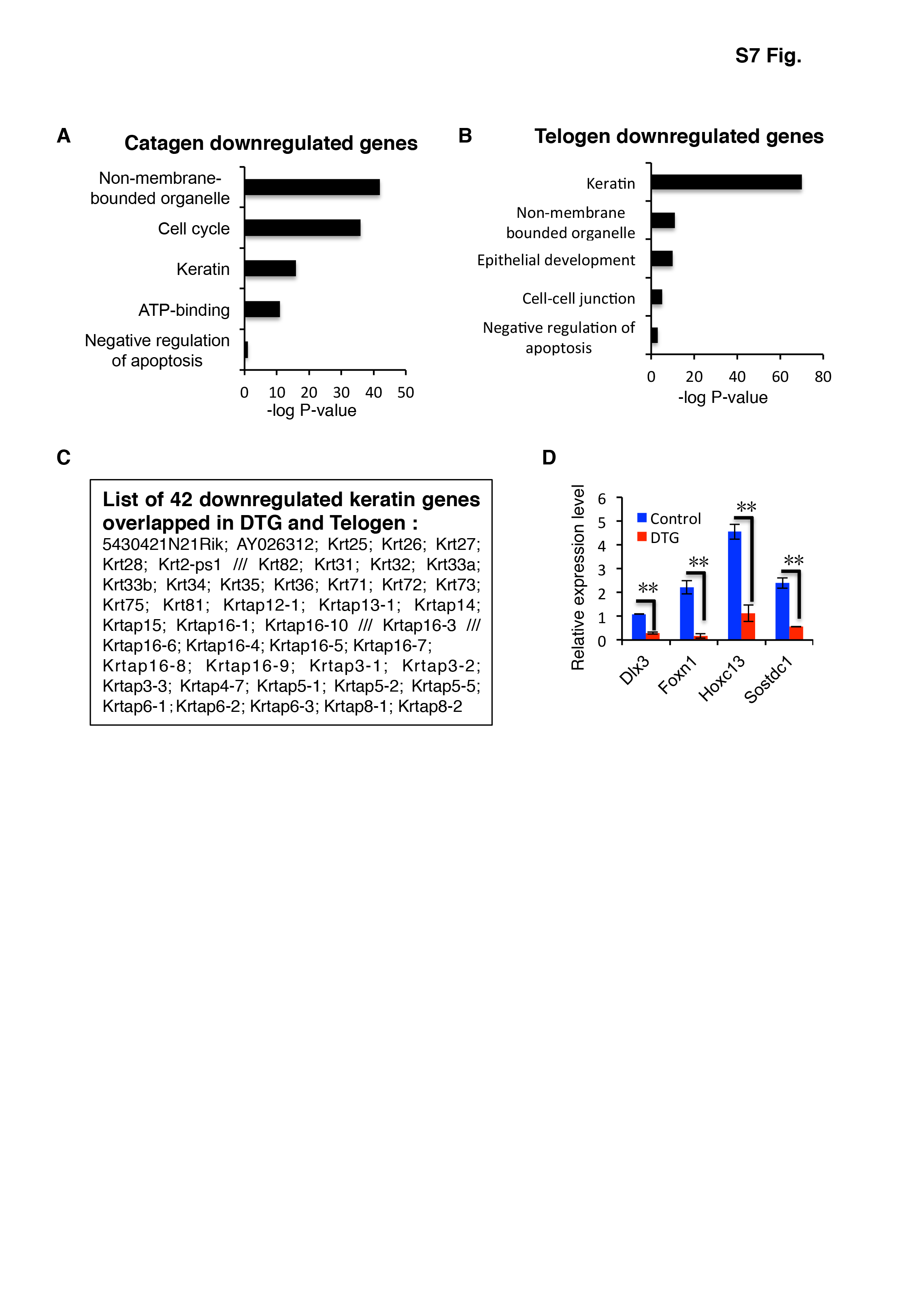

Supplement: S7 Fig — (A) Gene ontology analysis of downregulated genes in the catagen profiles. (B) Gene ontology analysis of downregulated genes in the telogen profiles. (C) List of common downregulated keratin genes in telogen and DTG profiles. (D) qPCR for Foxn1, Dlx3, Sostdc1 and Hoxc13 in control and DTG skin. ** p < 0.01. (TIF) [file pgen.1005253.s007.tif]

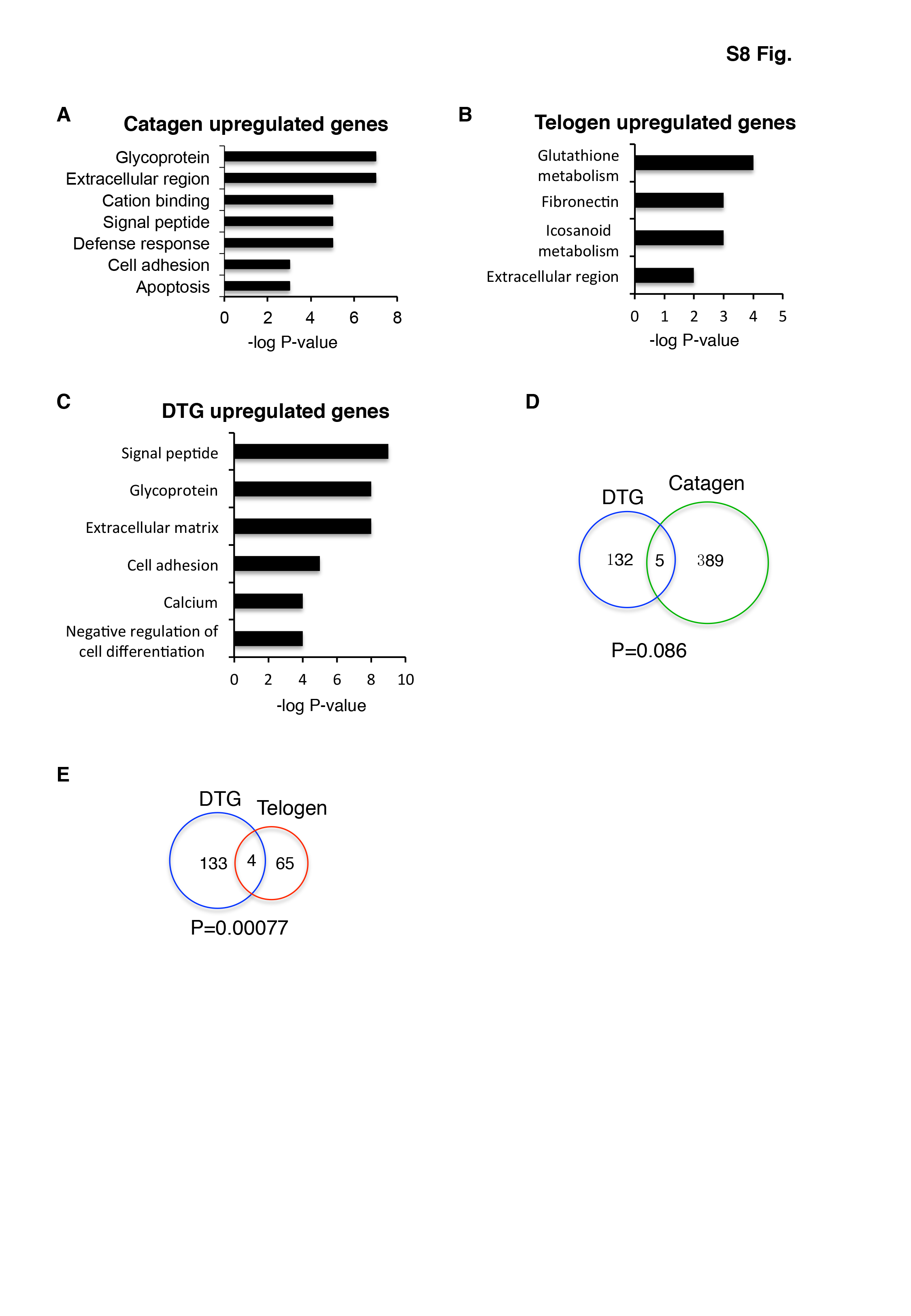

Supplement: S8 Fig — (A) Gene ontology analysis of upregulated genes in catagen. (B) Gene ontology analysis of upregulated genes in telogen. (C) Gene ontology analysis of upregulated genes in DTG skin. (D) Overlap of upregulated genes in catagen and DTG skin. (E) Overlap of downregulated genes in telogen and DTG skin. (TIF) [file pgen.1005253.s008.tif]

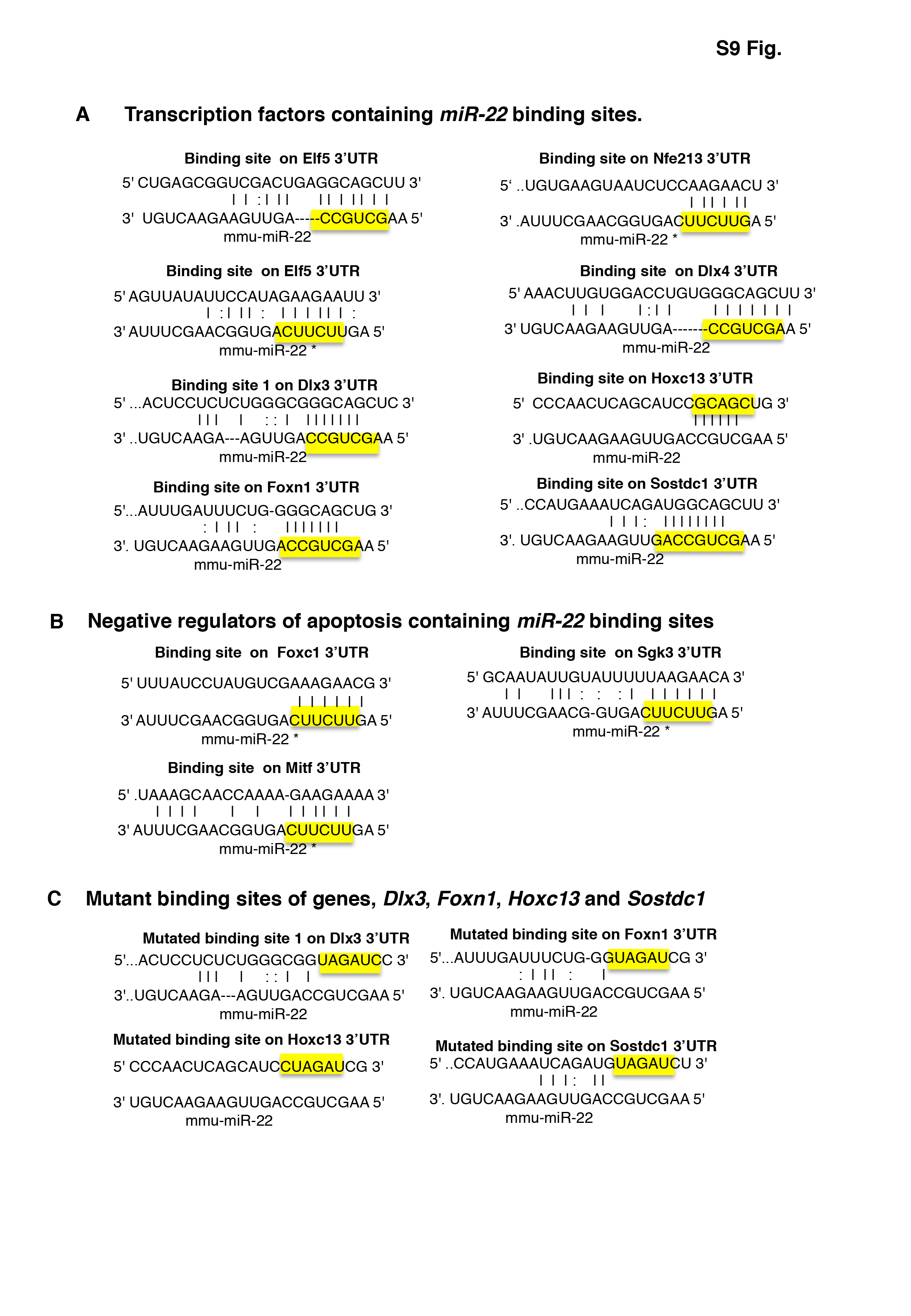

Supplement: S9 Fig — (A) The downregulated transcription factors containing miR-22 binding sites. (B) The downregulated negative regulators of apoptosis containing miR-22 binding sites. (C) Mutant binding sites of genes, Dlx3, Foxn1, Hoxc13 and Sostdc1. (TIF) [file pgen.1005253.s009.tif]

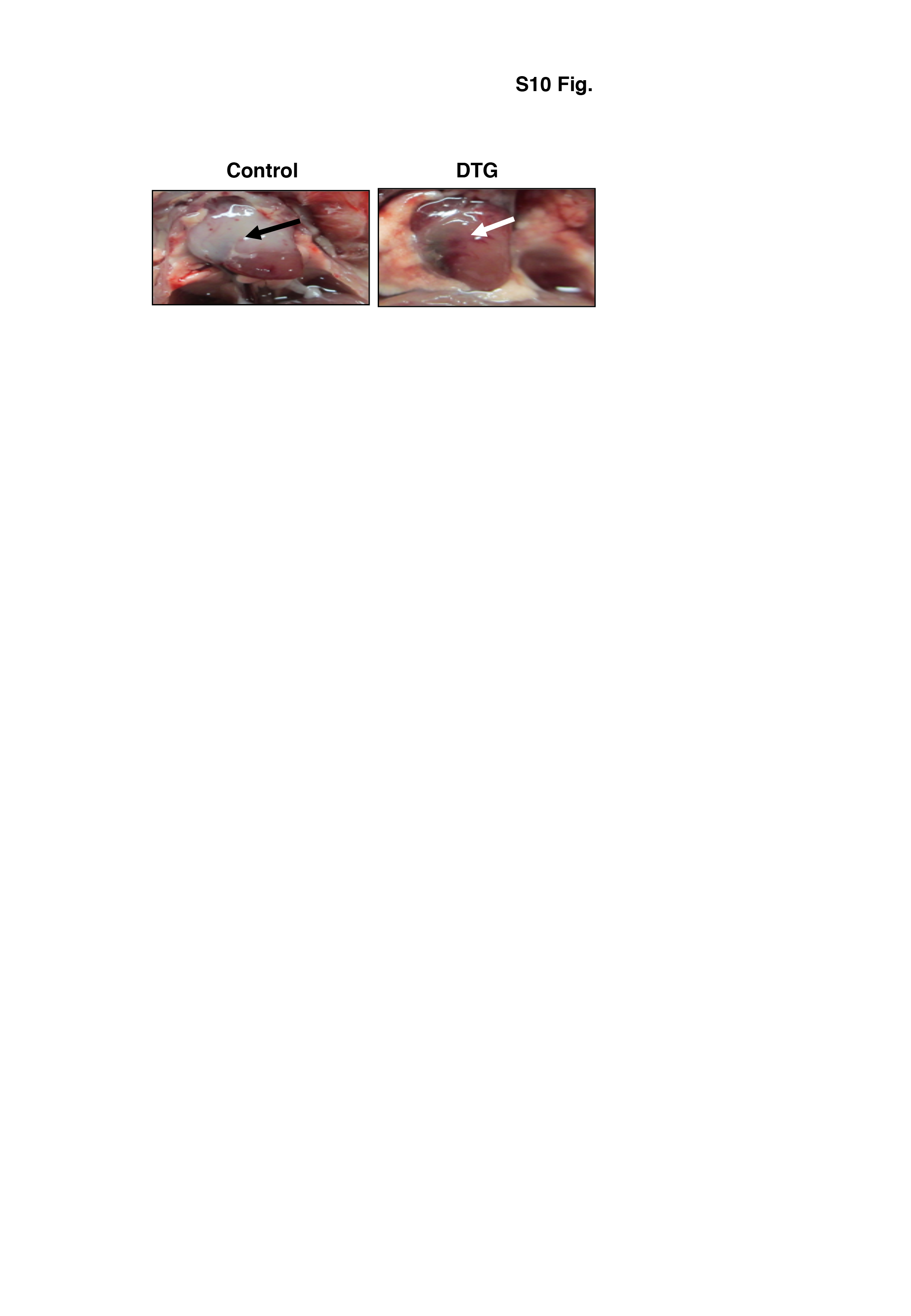

Supplement: S10 Fig — Gross pictures of thymus in the Control and DTG mice treated with Dox from P1 to P49. Arrows point to thymus. (TIF) [file pgen.1005253.s010.tif]
