# Supplementary material for: Post-transcriptional Regulation of Keratinocyte Progenitor Cell Expansion, Differentiation and Hair Follicle Regression by miR-22
Source: PLoS Genet. 2015 May 28;11(5):e1005253. doi: 10.1371/journal.pgen.1005253 (PMC4447420; doi:10.1371/journal.pgen.1005253)
Supplement: S1 Table — (PDF) [file pgen.1005253.s011.pdf]

S1 Table. List of 131 commonly downregulated genes between DTG and Catagen

Probe Set ID	Gene Symbol	q-value	Fold Change	Gene Title
1436398_at	---	0.0266675	0.5984	---
1440388_at	---	0	0.629	---
1452287_at	---	0.0338676	0.6075	---
1458880_at	---	0.0266675	0.6589	---
1447886_at	0610040B09Rik	0	0.4048	RIKEN cDNA 0610040B09 gene
1417803_at	1110032A04Rik	0	0.0428	RIKEN cDNA 1110032A04 gene
1430387_at	1810073O08Rik	0	0.4612	RIKEN cDNA 1810073O08 gene
1429909_at	2600010E01Rik	0.011749	0.6129	RIKEN cDNA 2600010E01 gene
1456210_at	5430407P10Rik	0	0.5192	RIKEN cDNA 5430407P10 gene
1427118_at	5430421N21Rik	0.0046807	0.084	RIKEN cDNA 5430421N21 gene
1457967_at	A030003K21Rik	0	0.0279	RIKEN cDNA A030003K21 gene
1422292_at	A030005K14Rik	0	0.0677	RIKEN cDNA A030005K14 gene
1421680_at	A030005L19Rik	0	0.025	RIKEN cDNA A030005L19 gene
1442425_at	A030014E15Rik	0	0.0198	RIKEN cDNA A030014E15 gene
1428273_at	Abhd13	0.0151567	0.6586	abhydrolase domain containing 13
1425559_a_at	Acsm3	0	0.4199	acyl-CoA synthetase medium-chain family member 3
1456901_at	Adamts20	0	0.3295	a disintegrin-like and metallopeptidase with thrombospondin type 1 motif, 20
1441094_at	Adh6b	0	0.1273	alcohol dehydrogenase 6B (class V)
1419549_at	Arg1	0.036665	0.5066	arginase, liver
1422929_s_at	Atoh7	0.0413137	0.4411	atonal homolog 7 (Drosophila)
1423753_at	Bambi	0	0.2969	BMP and activin membrane-bound inhibitor, homolog (Xenopus laevis)
1441991_at	BC039632	0	0.4402	cDNA sequence BC039632
1449907_at	Bcmo1	0.0046807	0.5019	beta-carotene 15,15'-monooxygenase
1449873_at	Bmp8a	0.0107895	0.5476	bone morphogenetic protein 8a
1419225_at	Cacna2d3	0	0.4987	calcium channel, voltage-dependent, alpha2/delta subunit 3
1449970_at	Capn12	0.0130118	0.5295	calpain 12
1425916_at	Capn8	0	0.1694	calpain 8
1428574_a_at	Chn2	0.0494965	0.6211	chimerin (chimaerin) 2
1449091_at	Cldn8	0.0046807	0.3918	claudin 8

1418599_at	Col11a1	0	0.5696	collagen, type XI, alpha 1
1450966_at	Crot	0	0.6021	carnitine O-octanoyltransferase
1420686_at	Cryba4	0	0.0992	crystallin, beta A4
1416776_at	Crym	0	0.1331	crystallin, mu
1423845_at	Csdc2	0.0429747	0.6213	cold shock domain containing C2, RNA binding
1416563_at	Ctps	0.0018723	0.5462	cytidine 5'-triphosphate synthase
1418989_at	Ctse	0	0.1791	cathepsin E
1439947_at	Cyp11a1	0.0046807	0.3693	cytochrome P450, family 11, subfamily a, polypeptide 1
1431171_at	D730001G18Rik	0	0.1079	RIKEN cDNA D730001G18 gene
1436532_at	Dclk3	0	0.3595	doublecortin-like kinase 3
1450475_at	Dlx3	0.0167986	0.6573	distal-less homeobox 3
1433596_at	Dnajc6	0	0.3656	DnaJ (Hsp40) homolog, subfamily C, member 6
1434297_at	E130304F04Rik	0.0046807	0.503	RIKEN cDNA E130304F04 gene
1419555_at	Elf5	0.0205577	0.4679	E74-like factor 5
1418569_at	Fblim1	0.0046807	0.6085	filamin binding LIM protein 1
1448470_at	Fbp1	0	0.2789	fructose bisphosphatase 1
1435551_at	Fhod3	0.0018723	0.4353	formin homology 2 domain containing 3
1456815_at	Foxn1	0.0046807	0.3623	forkhead box N1
1451424_at	Gabrp	0	0.1691	gamma-aminobutyric acid (GABA) A receptor, pi
1424296_at	Gclc	0.0167986	0.5938	glutamate-cysteine ligase, catalytic subunit
1439793_at	Gja3	0	0.1429	gap junction protein, alpha 3
1423271_at	Gjb2	0.0213976	0.6236	gap junction protein, beta 2
1417422_at	Gnmt	0	0.201	glycine N-methyltransferase
1420581_at	Gpr143	0.0231974	0.5293	G protein-coupled receptor 143
1450875_at	Gpr37	0.0167986	0.6059	G protein-coupled receptor 37
1437486_at	Gprc5a	0	0.5652	G protein-coupled receptor, family C, group 5, member A
1420538_at	Gprc5d	0	0.0458	G protein-coupled receptor, family C, group 5, member D
1435994_at	Kcnh1	0.0018723	0.4185	potassium voltage-gated channel, subfamily H (eag-related), member 1
1429913_at	Kcnk16	0.0250048	0.3823	potassium channel, subfamily K, member 16
1436160_at	Krt26	0	0.0604	keratin 26
1430132_at	Krt28	0.0046807	0.5623	keratin 28
1427719_s_at	Krt2-ps1 /// Krt82	0	0.0469	keratin complex 2, basic, pseudogene 1 /// keratin 82
1421589_at	Krt31	0	0.0472	keratin 31
1420728_at	Krt32	0	0.2079	keratin 32

1449387_at	Krt33a	0	0.0253	keratin 33A
1427179_at	Krt33b	0	0.0369	keratin 33B
1418742_at	Krt34	0	0.0174	keratin 34
1420409_at	Krt35	0.0071795	0.4303	keratin 35
1419840_at	Krt72	0	0.0796	keratin 72
1436557_at	Krt73	0.0046807	0.59	keratin 73
1427378_at	Krt75	0.0250048	0.4819	keratin 75
1427290_at	Krt81	0	0.0641	keratin 81
1450536_s_at	Krtap12-1	0	0.0563	keratin associated protein 12-1
1428007_at	Krtap13-1	0	0.025	keratin associated protein 13-1
1419707_at	Krtap14	0	0.0078	keratin associated protein 14
1419507_at	Krtap15	0	0.0033	keratin associated protein 15
1425655_at	Krtap16-1	0	0.0051	keratin associated protein 16-1
1427549_s_at	Krtap16-10 /// Krt	0	0.0026	keratin associated protein 16-10
1426203_at	Krtap16-4	0	0.0366	keratin associated protein 16-4
1425430_at	Krtap16-5	0	0.0058	keratin associated protein 16-5
1427800_at	Krtap16-9	0	0.1417	Keratin associated protein 16-9 (Krtap16-9), mRNA
1450539_at	Krtap5-1	0.0213976	0.2187	keratin associated protein 5-1
1420452_at	Krtap5-2	0	0.0268	keratin associated protein 5-2
1430728_at	Krtap5-5	0	0.0595	keratin associated protein 5-5
1421689_at	Krtap8-2	0	0.0059	keratin associated protein 8-2
1450774_at	Ly6g6d	0	0.0663	lymphocyte antigen 6 complex, locus G6D
1435461_at	Magi3	0.0046807	0.6671	membrane associated guanylate kinase, WW and PDZ domain containing 3
1435393_at	Mc1r	0	0.421	melanocortin 1 receptor
1448300_at	Mgst3	0.0089345	0.6046	microsomal glutathione S-transferase 3
1430635_at	Mlana	0	0.4554	melan-A
1449559_at	Msx2	0.0440131	0.5313	homeobox, msh-like 2
1450645_at	Mt4	0	0.1619	metallothionein 4
1456784_at	OTTMUSG0000(	0	0.5371	predicted gene, OTTMUSG00000015762
1417575_at	Otub2	0.0305899	0.6286	OTU domain, ubiquitin aldehyde binding 2
1428615_at	P2ry5	0.0122028	0.6538	purinergic receptor P2Y, G-protein coupled, 5
1419767_at	Padi3	0	0.2537	peptidyl arginine deiminase, type III
1426453_at	Pitrm1	0.0151567	0.6455	pitrilysin metallepetidase 1
1429183_at	Pkp2	0.0071795	0.6157	plakophilin 2

1420664_s_at	Procr	0.0122028	0.3414	protein C receptor, endothelial
1420467_at	Psors1c2	0	0.1408	psoriasis susceptibility 1 candidate 2 (human)
1429262_at	Rassf6	0.0322659	0.6173	Ras association (RalGDS/AF-6) domain family member 6
1425114_at	Rbbp6	0.011749	0.635	retinoblastoma binding protein 6
1434628_a_at	Rhpn2	0	0.294	rhophilin, Rho GTPase binding protein 2
1452734_at	Rnaset2a /// Rna	0	0.6528	ribonuclease T2A /// ribonuclease T2B
1429321_at	Rnf149	0.0046807	0.6612	ring finger protein 149
1421856_at	S100a3	0	0.1376	S100 calcium binding protein A3
1418805_at	Sct	0	0.3213	secretin
1450699_at	Selenbp1	0.0205577	0.4476	selenium binding protein 1
1424824_at	Slain1	0	0.4318	SLAIN motif family, member 1
1448502_at	Slc16a7	0.0321469	0.5923	solute carrier family 16 (monocarboxylic acid transporters), member 7
1417750_a_at	Slc25a37	0.0213976	0.5343	solute carrier family 25, member 37
1448566_at	Slc40a1	0	0.287	solute carrier family 40 (iron-regulated transporter), member 1
1437430_at	Slc45a2	0.0034597	0.3898	solute carrier family 45, member 2
1424338_at	Slc6a13	0	0.2614	solute carrier family 6 (neurotransmitter transporter, GABA), member 13
1455442_at	Slc6a19	0	0.4827	solute carrier family 6 (neurotransmitter transporter), member 19
1427787_at	Sp6	0.0305899	0.4611	trans-acting transcription factor 6
1449287_at	Srms	0.0046807	0.5707	src-related kinase lacking C-terminal tyrosine / N-terminal myristylation sites
1456147_at	St8sia6	0.0018723	0.3113	ST8 alpha-N-acetyl-neuraminide alpha-2,8-sialyltransferase 6
1424938_at	Steap1	0.0216197	0.5518	six transmembrane epithelial antigen of the prostate 1
1422723_at	Stra6	0.0466284	0.5343	stimulated by retinoic acid gene 6
1419289_a_at	Syngr1	0.0046807	0.6498	synaptogyrin 1
1421594_a_at	Sytl2	0.0135855	0.55	synaptotagmin-like 2
1427055_at	Them4	0	0.5753	thioesterase superfamily member 4
1450958_at	Tm4sf1	0.0046807	0.563	transmembrane 4 superfamily member 1
1418412_at	Tpd52l1	0.0206235	0.6362	tumor protein D52-like 1
1418935_at	Trpm1	0.0223754	0.5194	transient receptor potential cation channel, subfamily M, member 1
1460239_at	Tspan13	0.0223754	0.6691	tetraspanin 13
1448501_at	Tspan6	0	0.5038	tetraspanin 6
1455377_at	Ttll7	0.0034597	0.6254	tubulin tyrosine ligase-like family, member 7
1415978_at	Tubb3	0.0122028	0.5236	tubulin, beta 3
1417717_a_at	Tyr	0.0250048	0.5877	tyrosinase
1428307_at	Zdhhc13	0.0018723	0.6056	zinc finger, DHHC domain containing 13
