# Supplementary material for: Post-transcriptional Regulation of Keratinocyte Progenitor Cell Expansion, Differentiation and Hair Follicle Regression by miR-22
Source: PLoS Genet. 2015 May 28;11(5):e1005253. doi: 10.1371/journal.pgen.1005253 (PMC4447420; doi:10.1371/journal.pgen.1005253)
Supplement: S2 Table — (PDF) [file pgen.1005253.s012.pdf]

S2 Table. List of 153 commonly downregulated genes between DTG and Telogen

Probe Set ID	Gene Symbol	q-value	Fold Change	Gene Title
1439794_	---	0.021398	0.6495	---
1444768_	---	0	0.3062	---
1419032_	2610018G03Rik	0.018363	0.6201	RIKEN cDNA 2610018G03 gene
1455214_	Mitf	0.004878	0.6647	microphthalmia-associated transcription factor
1458467_	1110032D16Rik	0	0.0061	RIKEN cDNA 1110032D16 gene
1429835_	2310033E01Rik	0	0.1886	RIKEN cDNA 2310033E01 gene
1429960_	4930438A08Rik	0	0.3849	RIKEN cDNA 4930438A08 gene
1456210_	5430407P10Rik	0	0.5192	RIKEN cDNA 5430407P10 gene
1427118_	5430421N21Rik	0.004681	0.084	RIKEN cDNA 5430421N21 gene
1429896_	5830408B19Rik	0	0.5752	RIKEN cDNA 5830408B19 gene
1460517_	9130409J20Rik	0.025005	0.463	RIKEN cDNA 9130409J20 gene
1457967_	A030003K21Rik	0	0.0279	RIKEN cDNA A030003K21 gene
1422292_	A030005K14Rik	0	0.0677	RIKEN cDNA A030005K14 gene
1421680_	A030005L19Rik	0	0.025	RIKEN cDNA A030005L19 gene
1442425_	A030014E15Rik	0	0.0198	RIKEN cDNA A030014E15 gene
1428273_	Abhd13	0.015157	0.6586	abhydrolase domain containing 13
1425102_	Ace2	0	0.5152	angiotensin I converting enzyme (peptidyl-dipeptidase A) 2
1456901_	Adamts20	0	0.3295	a disintegrin-like metallopeptidase with thrombospondin type1 motif, 20
1456878_	AI646023	0	0.3429	expressed sequence AI646023
1425163_	AI661453	0.046628	0.6351	expressed sequence AI661453
1426300_	Alcam	0	0.6584	activated leukocyte cell adhesion molecule
1435154_	AU018091	0	0.1659	expressed sequence AU018091
1420415_	AY026312	0	0.0059	cDNA sequence AY026312
1423753_	Bambi	0	0.2969	BMP and activin membrane-bound inhibitor, homolog (Xenopus laevis)
1441991_	BC039632	0	0.4402	cDNA sequence BC039632
1449873_	Bmp8a	0.010789	0.5476	bone morphogenetic protein 8a
1419225_	Cacna2d3	0	0.4987	calcium channel, voltage-dependent, alpha2/delta subunit 3
1449970_	Capn12	0.013012	0.5295	calpain 12
1425916_	Capn8	0	0.1694	calpain 8
1448752_	Car2	0.027009	0.4018	carbonic anhydrase 2
1421001_	Car6	0.037391	0.2373	carbonic anhydrase 6
1439109_	Ccdc68	0.021398	0.5743	coiled-coil domain containing 68
1451382_	Chac1	0.044013	0.3079	ChaC, cation transport regulator-like 1 (E. coli)
1453009_	Cpm	0	0.3008	carboxypeptidase M

1420686_	Cryba4	0	0.0992	crystallin, beta A4
1416776_	Crym	0	0.1331	crystallin, mu
1423845_	Csdc2	0.042975	0.6213	cold shock domain containing C2, RNA binding
1418989_	Ctse	0	0.1791	cathepsin E
1418457_	Cxcl14	0.018767	0.648	chemokine (C-X-C motif) ligand 14
1428384_	D4Bwg0951e	0.004681	0.6115	DNA segment, Chr 4, Brigham & Women's Genetics 0951 expressed
1431171_	D730001G18Rik	0	0.1079	RIKEN cDNA D730001G18 gene
1452070_	Dedd2	0.025005	0.6432	death effector domain-containing DNA binding protein 2
1450475_	Dlx3	0.016799	0.6573	distal-less homeobox 3
1450698_	Dusp2	0	0.439	dual specificity phosphatase 2
1451924_	Edn1	0.042975	0.6592	endothelin 1
1419555_	Elf5	0.020558	0.4679	E74-like factor 5
1420751_	ENSMUSG0000	0	0.0041	predicted gene, ENSMUSG00000068074
1455188_	Ephb1	0	0.5028	Eph receptor B1
1449077_	Eraf	0.00346	0.2509	erythroid associated factor
1418773_	Fads3	0.022375	0.5249	fatty acid desaturase 3
1448470_	Fbp1	0	0.2789	fructose bisphosphatase 1
1460296_	Fgf22	0.020624	0.5266	fibroblast growth factor 22
1435551_	Fhod3	0.001872	0.4353	formin homology 2 domain containing 3
1445534_	Flnb	0.007179	0.6268	Filamin, beta, mRNA (cDNA clone IMAGE:3488025)
1453102_	Flrt3	0.03059	0.6657	fibronectin leucine rich transmembrane protein 3
1419485_	Foxc1	0.00346	0.6409	forkhead box C1
1456815_	Foxn1	0.004681	0.3623	forkhead box N1
1418207_	Fxyd4	0.03059	0.295	FXYD domain-containing ion transport regulator 4
1451424_	Gabrp	0	0.1691	gamma-aminobutyric acid (GABA) A receptor, pi
1424296_	Gclc	0.016799	0.5938	glutamate-cysteine ligase, catalytic subunit
1439793_	Gja3	0	0.1429	gap junction protein, alpha 3
1423271_	Gjb2	0.021398	0.6236	gap junction protein, beta 2
1420538_	Gprc5d	0	0.0458	G protein-coupled receptor, family C, group 5, member D
1439566_	Gprin3	0.03059	0.6105	GPRIN family member 3
1425874_	Hoxc13	0.008934	0.5011	homeo box C13
1420640_	Jmy	0.020624	0.5515	junction-mediating and regulatory protein
1429913_	Kcnk16	0.025005	0.3823	potassium channel, subfamily K, member 16
1418173_	Krt25	0.022375	0.5715	keratin 25
1436160_	Krt26	0	0.0604	keratin 26
1449378_	Krt27	0.00346	0.3423	keratin 27
1430132_	Krt28	0.004681	0.5623	keratin 28
1427719_	Krt2-ps1 /// Krt82	0	0.0469	keratin complex 2, basic, pseudogene 1 /// keratin 82
1421589_	Krt31	0	0.0472	keratin 31

1420728_	Krt32	0	0.2079	keratin 32
1449387_	Krt33a	0	0.0253	keratin 33A
1427179_	Krt33b	0	0.0369	keratin 33B
1418742_	Krt34	0	0.0174	keratin 34
1420409_	Krt35	0.007179	0.4303	keratin 35
1427751_	Krt36	0.004681	0.345	keratin 36
1448457_	Krt71	0.025005	0.45	keratin 71
1419840_	Krt72	0	0.0796	keratin 72
1436557_	Krt73	0.004681	0.59	keratin 73
1427378_	Krt75	0.025005	0.4819	keratin 75
1427290_	Krt81	0	0.0641	keratin 81
1450536_	Krtap12-1	0	0.0563	keratin associated protein 12-1
1428007_	Krtap13-1	0	0.025	keratin associated protein 13-1
1419707_	Krtap14	0	0.0078	keratin associated protein 14
1419507_	Krtap15	0	0.0033	keratin associated protein 15
1425655_	Krtap16-1	0	0.0051	keratin associated protein 16-1
1427549_	Krtap16-10 /// Kr	0	0.0026	keratin associated protein 16-10
1426203_	Krtap16-4	0	0.0366	keratin associated protein 16-4
1425430_	Krtap16-5	0	0.0058	keratin associated protein 16-5
1421691_	Krtap16-7	0	0.0116	keratin associated protein 16-7
1425237_	Krtap16-8	0	0.0024	keratin associated protein 16-8
1427800_	Krtap16-9	0	0.1417	Keratin associated protein 16-9 (Krtap16-9), mRNA
1427366_	Krtap3-1	0.004681	0.1458	keratin associated protein 3-1
1430625_	Krtap3-2	0.02162	0.322	keratin associated protein 3-2
1452957_	Krtap3-3	0	0.0747	keratin associated protein 3-3
1430669_	Krtap4-7	0	0.0166	keratin associated protein 4-7
1450539_	Krtap5-1	0.021398	0.2187	keratin associated protein 5-1
1420452_	Krtap5-2	0	0.0268	keratin associated protein 5-2
1430728_	Krtap5-5	0	0.0595	keratin associated protein 5-5
1451859_	Krtap6-1	0	0.0046	keratin associated protein 6-1
1449919_	Krtap6-2	0	0.0146	keratin associated protein 6-2
1427842_	Krtap6-3	0	0.0152	keratin associated protein 6-3
1427211_	Krtap8-1	0	0.0137	keratin associated protein 8-1
1421689_	Krtap8-2	0	0.0059	keratin associated protein 8-2
1450774_	Ly6g6d	0	0.0663	lymphocyte antigen 6 complex, locus G6D
1441481_	Mfap3l	0.049496	0.6572	microfibrillar-associated protein 3-like
1423265_	Minpp1	0.004681	0.5762	multiple inositol polyphosphate histidine phosphatase 1
1422975_	Mme	0	0.4674	membrane metallo endopeptidase
1449559_	Msx2	0.044013	0.5313	homeobox, msh-like 2

1417520_	Nfe2l3	0.00346	0.5737	nuclear factor, erythroid derived 2, like 3
1422790_	Nppc	0.018767	0.365	natriuretic peptide precursor type C
1443043_	Otop2	0	0.2247	otopetrin 2
1456784_	OTTMUSG0000(	0	0.5371	predicted gene, OTTMUSG00000015762
1417575_	Otub2	0.03059	0.6286	OTU domain, ubiquitin aldehyde binding 2
1419323_	Padi1	0.004681	0.2153	peptidyl arginine deiminase, type I
1419767_	Padi3	0	0.2537	peptidyl arginine deiminase, type III
1453283_	Pgm1	0.004681	0.5432	phosphoglucomutase 1
1435462_	Plcxd2	0.020558	0.5141	phosphatidylinositol-specific phospholipase C, X domain containing 2
1447807_	Plekhh1	0.016799	0.6692	pleckstrin homology domain containing, familyH (with MyTH4 domain) member1
1420467_	Psors1c2	0	0.1408	psoriasis susceptibility 1 candidate 2 (human)
1429262_	Rassf6	0.032266	0.6173	Ras association (RalGDS/AF-6) domain family member 6
1425114_	Rbbp6	0.011749	0.635	retinoblastoma binding protein 6
1434628_	Rhpn2	0	0.294	rhophilin, Rho GTPase binding protein 2
1429321_	Rnf149	0.004681	0.6612	ring finger protein 149
1441793_	Rnf39	0.026668	0.6272	Ring finger protein 39 (Rnf39), mRNA
1419814_	S100a1	0	0.6379	S100 calcium binding protein A1
1421856_	S100a3	0	0.1376	S100 calcium binding protein A3
1420764_	Scrg1	0.004878	0.538	scrapie responsive gene 1
1420918_	Sgk3	0.001872	0.6105	serum/glucocorticoid regulated kinase 3
1424824_	Slain1	0	0.4318	SLAIN motif family, member 1
1417750_	Slc25a37	0.021398	0.5343	solute carrier family 25, member 37
1448566_	Slc40a1	0	0.287	solute carrier family 40 (iron-regulated transporter), member 1
1416464_	Slc4a1	0.031133	0.4146	solute carrier family 4 (anion exchanger), member 1
1455442_	Slc6a19	0	0.4827	solute carrier family 6 (neurotransmitter transporter), member 19
1417929_	Slc7a8	0.012203	0.572	solute carrier family 7 (cationic amino acid transporter, y+ system), member 8
1460616_	Slco4c1	0.00346	0.3654	solute carrier organic anion transporter family, member 4C1
1456883_	Stox1	0.004681	0.2466	storkhead box 1
1421594_	Sytl2	0.013586	0.55	synaptotagmin-like 2
1434425_	Tchh	0	0.3016	trichohyalin
1429944_	Tchhl1	0	0.0176	trichohyalin-like 1
1437702_	Tgm6	0	0.5019	transglutaminase 6
1450958_	Tm4sf1	0.004681	0.563	transmembrane 4 superfamily member 1
1454709_	Tmem64	0.013012	0.67	transmembrane protein 64
1458871_	Tmprss11e	0.004681	0.5132	transmembrane protease, serine 11e
1456623_	Tpm1	0.013012	0.6434	tropomyosin 1, alpha
1419615_	Trpv6	0.025005	0.5695	transient receptor potential cation channel, subfamily V, member 6
1453269_	Unc5b	0.015157	0.6681	unc-5 homolog B (C. elegans)
1448562_	Upp1	0	0.2207	uridine phosphorylase 1

1428307_	Zdhhc13	0.001872	0.6056	zinc finger, DHHC domain containing 13
1420944_	Zfp185	0.02162	0.6231	zinc finger protein 185
