# Supplementary material for: Post-transcriptional Regulation of Keratinocyte Progenitor Cell Expansion, Differentiation and Hair Follicle Regression by miR-22
Source: PLoS Genet. 2015 May 28;11(5):e1005253. doi: 10.1371/journal.pgen.1005253 (PMC4447420; doi:10.1371/journal.pgen.1005253)
Supplement: S3 Table — (PDF) [file pgen.1005253.s013.pdf]

S3 Table. List of 74 commonly downregulated genes among DTG, Catagen and Telogen

Probe Set ID	Gene Symbol	q-value	Fold Change	Gene Title
1456210_	5430407P10Rik	0	0.5192	RIKEN cDNA 5430407P10 gene
1427118_	5430421N21Rik	0.0047	0.084	RIKEN cDNA 5430421N21 gene
1457967_	A030003K21Rik	0	0.0279	RIKEN cDNA A030003K21 gene
1422292_	A030005K14Rik	0	0.0677	RIKEN cDNA A030005K14 gene
1421680_	A030005L19Rik	0	0.025	RIKEN cDNA A030005L19 gene
1442425_	A030014E15Rik	0	0.0198	RIKEN cDNA A030014E15 gene
1428273_	Abhd13	0.0152	0.6586	abhydrolase domain containing 13
1456901_	Adamts20	0	0.3295	a disintegrin-like and metallopeptidase with thrombospondin type 1 motif, 20
1423753_	Bambi	0	0.2969	BMP and activin membrane-bound inhibitor, homolog (Xenopus laevis)
1441991_	BC039632	0	0.4402	cDNA sequence BC039632
1449873_	Bmp8a	0.0108	0.5476	bone morphogenetic protein 8a
1419225_	Cacna2d3	0	0.4987	calcium channel, voltage-dependent, alpha2/delta subunit 3
1449970_	Capn12	0.013	0.5295	calpain 12
1425916_	Capn8	0	0.1694	calpain 8
1420686_	Cryba4	0	0.0992	crystallin, beta A4
1416776_	Crym	0	0.1331	crystallin, mu
1423845_	Csdc2	0.043	0.6213	cold shock domain containing C2, RNA binding
1418989_	Ctse	0	0.1791	cathepsin E
1431171_	D730001G18Rik	0	0.1079	RIKEN cDNA D730001G18 gene
1450475_	Dlx3	0.0168	0.6573	distal-less homeobox 3
1419555_	Elf5	0.0206	0.4679	E74-like factor 5
1448470_	Fbp1	0	0.2789	fructose bisphosphatase 1
1435551_	Fhod3	0.0019	0.4353	formin homology 2 domain containing 3
1456815_	Foxn1	0.0047	0.3623	forkhead box N1
1451424_	Gabrp	0	0.1691	gamma-aminobutyric acid (GABA) A receptor, pi
1424296_	Gclc	0.0168	0.5938	glutamate-cysteine ligase, catalytic subunit
1439793_	Gja3	0	0.1429	gap junction protein, alpha 3
1423271_	Gjb2	0.0214	0.6236	gap junction protein, beta 2
1420538_	Gprc5d	0	0.0458	G protein-coupled receptor, family C, group 5, member D
1429913_	Kcnk16	0.025	0.3823	potassium channel, subfamily K, member 16
1436160_	Krt26	0	0.0604	keratin 26
1430132_	Krt28	0.0047	0.5623	keratin 28
1427719_	Krt2-ps1 /// Krt82	0	0.0469	keratin complex 2, basic, pseudogene 1 /// keratin 82

1421589_	Krt31	0	0.0472	keratin 31
1420728_	Krt32	0	0.2079	keratin 32
1449387_	Krt33a	0	0.0253	keratin 33A
1427179_	Krt33b	0	0.0369	keratin 33B
1418742_	Krt34	0	0.0174	keratin 34
1420409_	Krt35	0.0072	0.4303	keratin 35
1419840_	Krt72	0	0.0796	keratin 72
1436557_	Krt73	0.0047	0.59	keratin 73
1427378_	Krt75	0.025	0.4819	keratin 75
1427290_	Krt81	0	0.0641	keratin 81
1450536_	Krtap12-1	0	0.0563	keratin associated protein 12-1
1428007_	Krtap13-1	0	0.025	keratin associated protein 13-1
1419707_	Krtap14	0	0.0078	keratin associated protein 14
1419507_	Krtap15	0	0.0033	keratin associated protein 15
1425655_	Krtap16-1	0	0.0051	keratin associated protein 16-1
1427549_	Krtap16-10 /// Krt	0	0.0026	keratin associated protein 16-10
1426203_	Krtap16-4	0	0.0366	keratin associated protein 16-4
1425430_	Krtap16-5	0	0.0058	keratin associated protein 16-5
1427800_	Krtap16-9	0	0.1417	Keratin associated protein 16-9 (Krtap16-9), mRNA
1450539_	Krtap5-1	0.0214	0.2187	keratin associated protein 5-1
1420452_	Krtap5-2	0	0.0268	keratin associated protein 5-2
1430728_	Krtap5-5	0	0.0595	keratin associated protein 5-5
1421689_	Krtap8-2	0	0.0059	keratin associated protein 8-2
1450774_	Ly6g6d	0	0.0663	lymphocyte antigen 6 complex, locus G6D
1449559_	Msx2	0.044	0.5313	homeobox, msh-like 2
1456784_	OTTMUSG0000(	0	0.5371	predicted gene, OTTMUSG00000015762
1417575_	Otub2	0.0306	0.6286	OTU domain, ubiquitin aldehyde binding 2
1419767_	Padi3	0	0.2537	peptidyl arginine deiminase, type III
1420467_	Psors1c2	0	0.1408	psoriasis susceptibility 1 candidate 2 (human)
1429262_	Rassf6	0.0323	0.6173	Ras association (RalGDS/AF-6) domain family member 6
1425114_	Rbbp6	0.0117	0.635	retinoblastoma binding protein 6
1434628_	Rhpn2	0	0.294	rhophilin, Rho GTPase binding protein 2
1429321_	Rnf149	0.0047	0.6612	ring finger protein 149
1421856_	S100a3	0	0.1376	S100 calcium binding protein A3
1424824_	Slain1	0	0.4318	SLAIN motif family, member 1
1417750_	Slc25a37	0.0214	0.5343	solute carrier family 25, member 37
1448566_	Slc40a1	0	0.287	solute carrier family 40 (iron-regulated transporter), member 1
1455442_	Slc6a19	0	0.4827	solute carrier family 6 (neurotransmitter transporter), member 19
1421594_	Sytl2	0.0136	0.55	synaptotagmin-like 2

1450958_	Tm4sf1	0.0047	0.563	transmembrane 4 superfamily member 1
1428307_	Zdhhc13	0.0019	0.6056	zinc finger, DHHC domain containing 13
