# Supplementary material for: Post-transcriptional Regulation of Keratinocyte Progenitor Cell Expansion, Differentiation and Hair Follicle Regression by miR-22
Source: PLoS Genet. 2015 May 28;11(5):e1005253. doi: 10.1371/journal.pgen.1005253 (PMC4447420; doi:10.1371/journal.pgen.1005253)
Supplement: S4 Table — (PDF) [file pgen.1005253.s014.pdf]

S4 Table. The sequences of primer used in this paper

Primer Name	Sequence (5' to 3')
Dlx3-forward	GTGCCTTAGGGGTAAGGCTGTCAG
Dlx3-reverse	GGGACCTGCTTCTCTTGGTTGCTC
Foxn1-forward	GGCCCTCAATCCTTCCAAAATCGAC
Foxn1-reverse	GCTGGATGCATTGGGTG-CAGAGG
Hoxc13-forward	TAAAGGAGATAGAGGGTGGGTCCCT
Hoxc13-reverse	CCACAGCACCAAGTTGGCTTTTGAG
Sostdc1-forward	CCCAAACTAAATCCCTCCGAACCAG
Sostdc1-reverse	GCAGG-GGGATAATTTCACACTGAG
K32-forward	GGAGTGTGAGATTGACACGTACAG
K32-reverse	CTTCAGTAGCGGGTCTGTAGGCA
K35-forward	GCAGTGCATGATCGGCAA-TGTGG
K35-reverse	GGTACGAGCTGCGCCCGTGCT
K85-forward	CCTGGGTCAAGGCA-TGATCCTAA
K85-reverse	AGAGTAGTTTGCCCCAAAGGAA
K17-forward	GGAGATCGCC-ACCTACCGCCGTC
K17-reverse	GTGATGCCGGAGCGGAGAGGGAAG
MMP11-forward	GCGGTTGGGAGTGGTGTTGGTAAT
MMP11-reverse	GGCAAGGCTGTGAGGTATGTG
TGFB2- forward	GGATCCATGAACCCAAAGGGTAC
TGFB2-reverse	GCTGTTCGATCTTGGGCGTAT
K16-forward	CCTTGGAGAACAGCCTAGAAG
K16-reverse	ATTCTCGCCATCCAGCAGAC
Spink12-forward	GCAGCAACTATGAGAAGACACTGG
Spink12-reverse	TTGGTGGGAAGCAC-ACTGCTGTGG
Dlx3-F1	CCCTCGAGTGAGGGAACCTTTGATATCC
Dlx3-R1	GCGGCCGCACATAAATAGGAAACCACAA
Foxn1-F1	GCGATCGCGCAGGTGAACTGGCACCTCC
Foxn1-R1	GCGGCCGCCAAGTCTGTAGAGCACATGG
Hoxc13-F1	AGCTCGAGCGAACCTCTCGGAGCGTCAG
Hoxc13-R1	GCGGCCGCACATTACACTGCCAGGCCCT
Sostdc1-F1	GCCTCGAGCTAAATCCCTCCGAACCAGA
Sostdc1-R1	GCGGCCGCCAATGTCTGCAAGCATGTAC
